# Tumor-suppressive function of EZH2 is through inhibiting glutaminase

**DOI:** 10.1038/s41419-021-04212-7

**Published:** 2021-10-20

**Authors:** Yongfeng Liu, Cheng-e Tu, Xuxue Guo, Changjie Wu, Chuncai Gu, Qiuhua Lai, Yuxin Fang, Junqi Huang, Zhizhang Wang, Aimin Li, Side Liu

**Affiliations:** 1 Guangdong Provincial Key Laboratory of Gastroenterology, Department of Gastroenterology, Nanfang Hospital, Southern Medical University, Guangzhou, 510515 China; 2 Laboratory for Regenerative Medicine, Ministry of Education, College of Life Science and Technology, Jinan University, Guangzhou, 510632 China; 3 Department of Pathology, Nanfang Hospital, Southern Medical University, Guangzhou, 510515 China

**Keywords:** Cancer metabolism, Colon cancer

## Abstract

Tumors can use metabolic reprogramming to survive nutrient stress. Epigenetic regulators play a critical role in metabolic adaptation. Here we screened a sgRNA library to identify epigenetic regulators responsible for the vulnerability of colorectal cancer (CRC) cells to glucose deprivation and found that more EZH2-knockout cells survived glucose deprivation. Then, we showed that EZH2 expression was significantly downregulated in response to glucose deprivation in a glucose-sensitive CRC cell line, and EZH2-knockdown cells were more resistant to glucose deprivation. Mechanistically, EZH2 deficiency upregulated the expression of glutaminase (GLS) and promoted the production of glutamate, which in turn led to increased synthesis of intracellular glutathione (GSH) and eventually attenuated the reactive oxygen species (ROS)-mediated cell death induced by glucose deprivation. Although EZH2 functioned as an oncogene in cancer progression and EZH2 knockout abolished colorectal cancer development in a mouse model, here we revealed a mechanistic link between EZH2 and metabolic reprogramming via the direct regulation of *GLS* expression and observed a negative correlation between EZH2 and GLS expression in colorectal cancer tissues. These findings further confirmed the importance of heterogeneity, provided an explanation for the clinical tolerance of cancer cells to EZH2 inhibitors from the perspective of metabolism, and proposed the possibility of combining EZH2 inhibitors and glutamine metabolism inhibitors for the treatment of cancer.

## Introduction

Studies of metabolic vulnerability have identified many therapeutic targets for the treatment of cancer [1, 2]. During cancer progression, highly proliferative cancer cells are frequently exposed to nutrient-scarce conditions that result from poor vascularization or antiangiogenic therapy; thus, cancer cells need to adapt to nutrient stress, including the stress caused by glucose limitation. Cancer cells transformed by oncogenes, such as MYC, often display glucose addiction, which makes them highly dependent on glucose metabolism for growth and redox homeostasis [3–5], and the exhaustion of glucose in the microenvironment disrupts the intracellular homeostasis of cancer cells, ultimately leading to cell death. The regulation of glucose metabolism has been thoroughly studied in previous research, and more factors that promote glucose addiction have been identified, but factors responsible for adaptation to glucose limitation are relatively rare.

Covalent modifications of histones, including methylation and acetylation, play fundamental roles in the epigenetic regulation of gene expression. Histone methylation can either activate or repress gene transcription, depending on the position of the lysine residue within the histone and the combination of different modifications. Recent genomic studies have enhanced the understanding of the role of histone modifiers in cancer progression [6, 7]. However, the particular epigenetic regulators involved in the adaptation to glucose limitation have rarely been studied.

To study the epigenetic regulation of cancer cells under glucose deprivation conditions, we screened a knockout library of histone modifiers in the glucose-sensitive CRC cell line HCT116. First, the infected HCT116 cells were cultured in a glucose-depleted medium as indicated, and then, the dead and live cells were separated for gRNA sequencing. The results showed that gRNAs that target EZH2 were enriched in the surviving cells, which suggested that EZH2 might be involved in the cancer cell response to glucose deprivation. EZH2 is the enzymatic subunit of polycomb repressive complex 2 (PRC2), which tri-methylates H3K27 and facilitates chromatin compaction and gene silencing [8]. As an oncogene in solid cancers, EZH2 has been reported to affect many cancer-related signaling pathways and eventually promote cancer cell proliferation, malignant transformation, and survival [9–12].

Mechanistically, we identified *glutaminase* (*GLS*) as the direct target gene of EZH2 and showed that its expression could be silenced by EZH2 under glucose deprivation conditions. When the function of EZH2 is blocked with shRNA or inhibitors, the repression of *GLS* transcription is alleviated, and high GLS expression accelerates glutamine metabolism and GSH accumulation, which in turn attenuates glucose-deprivation-induced reactive oxygen species (ROS) production and cell death. In summary, these data suggest a tumor-suppressive role of EZH2 under glucose deprivation conditions.

## Results

### Conditional screening reveals that EZH2 is involved in the adaptation to glucose starvation

**Fig. 1:**
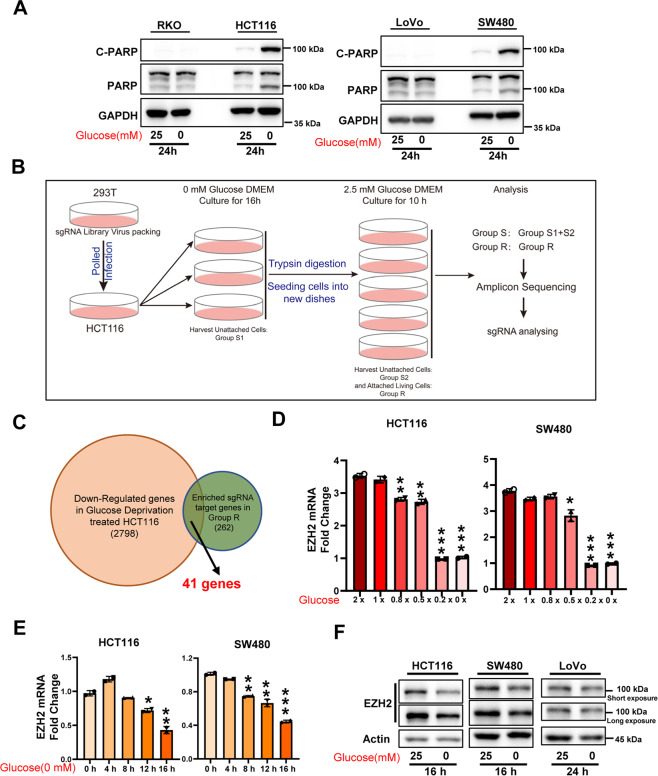
Library screening shows that EZH2 expression is regulated by glucose supply. **A** Immunoblotting for cleaved-PARP and PARP expression in indicated colorectal cancer (CRC) cells under glucose deprivation. GAPDH is used as a loading control. **B** Schematic diagram of library screening under glucose deprivation. **C** Venn diagram shows the overlap between genes downregulated by glucose deprivation treatment (*n* = 2798), and genes targeted by sgRNAs that enriched in Group R (*n* = 262). **D**, **E** RT-qPCR of *EZH2* levels in HCT116 and SW480 cells treated with indicated doses of glucose for 24 h (**D**) or under glucose deprivation for the indicated time (**E**). 1 × glucose, 25 mM. **F** Immunoblotting for EZH2 expression in indicated CRC cells under glucose deprivation. Actin is used as a loading control. The experiments in **A**, **F** were repeated twice independently with similar results. In **D**, **E**, data are mean ± s.d., *n* = 3 independent experiments; *P*-values were calculated using two-tailed unpaired Student’s *t*-test. *P < 0.05, **P < 0.01, ***P < 0.001.

Increasing evidence shows that metabolic reprogramming is an important adaptive mechanism that facilitates the survival and proliferation of cancer cells in nutrient-deficient tumor microenvironments [13], and epigenetic regulators play a critical role in this mechanism [14]. First, we investigated the sensitivity of different CRC cell lines to glucose starvation. The results showed that glucose starvation induced significant cell death, as indicated by the levels of cleaved PARP (C-PARP), only in glucose-sensitive cell lines (such as HCT116 and SW480 cells) but not in RKO and LoVo cells (Fig. 1A). Therefore, to identify the epigenetic factors responsible for adaptation to metabolic stress during glucose deprivation, we conducted genetic screening in HCT116 cells by using a sgRNA library targeting histone modifiers, including “writers”, “erasers”, and “readers” of acetylation and methylation (Fig. 1B, Supplementary Table 1). HCT116 cells were infected with the library, and cultured without glucose for 16 h. The suspended dead cells were harvested, and the rest cells were digested by trypsin and re-cultured in DMEM supplemented with 2.5 mM glucose for another 10 h to further separate dead cells attached to live cells. Then the cells were separated into two groups: the unattached dead cells (group S, sensitive to glucose deprivation) and the attached live cells (group R, resistance to glucose deprivation). The separated cells were subjected to genomic DNA isolation and deep sequencing of the integrated sgRNAs. After analysis, 262 candidate gene sgRNAs were enriched in group R compared with group S (fold change >1.5) (Supplementary Table 2). By comparing these candidate genes with the downregulated genes in HCT116 cells exposed to glucose deprivation (2798 genes), we obtained a list of 41 genes, among which EZH2 stood out (Fig. 1C and Supplementary Table 3). Further analysis showed that sgRNAs that target other subunits of polycomb repressive complex 2 (PRC2), such as EED, SUZ12, and PHF19, also tended to be enriched in group R (Supplementary Fig. 1A). These results led us to wonder whether the expression of EZH2 in HCT116 cells could be downregulated to adapt to glucose deprivation. To test this idea, CRC cells were cultured with or without glucose, and the expression of EZH2, SUZ12, and EED was detected. The results showed that glucose deprivation decreased EZH2 mRNA levels in a dose- and time-dependent manner in the glucose-sensitive CRC cell lines HCT116 and SW480 (Fig. 1D, E and Supplementary Fig. 1B, C) but not in the glucose-insensitive cell lines RKO and LoVo (Supplementary Fig. 1D, E). Additionally, similar results were obtained when the EZH2 protein levels were examined (Fig. 1F and Supplementary Fig. 2B). These data suggest that downregulation of EZH2 expression in glucose-sensitive CRC cells may be a strategy by which some cancer cells adapt to glucose deprivation.

### Downregulation of EZH2 expression alleviates glucose dependence in glucose-sensitive CRC cells

**Fig. 2:**
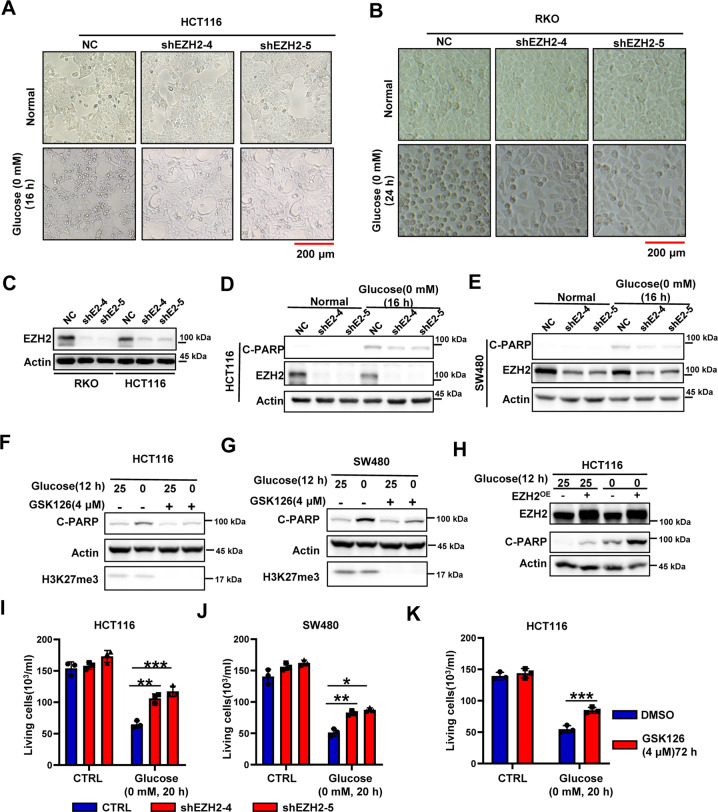
Absence of EZH2 in glucose-sensitive CRCs enhanced the tolerance to glucose-deprivation-induced cell death. **A**, **B** Representative images of HCT116 (**A**) or RKO (**B**) NC and shEZH2 cells under normal condition or glucose deprivation. The experiments were repeated twice independently with similar results. **C** Immunoblotting for EZH2 knockdown efficiency in RKO and HCT116 cells. Actin is used as a loading control. **D**, **E** Immunoblotting for cleaved-PARP and EZH2 expression in HCT116 (**D**) or SW480 (**E**) NC and shEZH2 cells under normal condition or glucose deprivation (0 mM, 16 h). **F**, **G** Immunoblotting for cleaved-PARP and H3K27me3 expression in HCT116 (**F**) or SW480 (**G**) cells pretreated with or without GSK126 (4 μM, 48 h), followed by glucose deprivation treatment (0 mM, 12 h). **H** Immunoblotting for cleaved-PARP and EZH2 expression in HCT116 cells with or without EZH2 overexpression under normal condition or glucose deprivation. **I**, **J** Living cell counting of HCT116 (**I**) or SW480 (**J**) NC and shEZH2 cells under normal condition or glucose deprivation (0 mM, 20 h). **K** Living cell counting of HCT116 cells pretreated with or without GSK126 (4 μM, 72 h), cultured under normal condition or glucose deprivation (0 mM, 20 h). The experiments in **C–H** were repeated twice independently with similar results. In **I**, **J**, **K**, data are mean ± s.d., *n* = 3 independent experiments; *P*-values were calculated using two-tailed unpaired Student’s *t*-test. *P < 0.05, **P < 0.01, ***P < 0.001. Scale bars, 200 μm.

After our previous work on the function of EZH2 in colitis [15], we conducted related studies on the function of EZH2 in colorectal cancer. Using the APC^min/+^ and AOM/DSS models, we showed that the deletion of the EZH2 gene significantly inhibited the occurrence of colon cancer, indicating a cancer-promoting function of EZH2 in CRC (Supplementary Fig. 3A–H); these results were consistent with the function of EZH2 in other solid cancers [11, 16, 17]. However, the paradoxical results in the screening described above suggested that EZH2 might perform different functions under stressful conditions, such as glucose deprivation, and made us wonder whether EZH2 could play an unconventional role in metabolism. To test this possibility, we generated EZH2-knockdown CRC cells and cultured them a in glucose-free medium. Observations of cell morphology revealed that the cell death in HCT116 cells and the cell cycle arrest in RKO cells caused by glucose deprivation were both attenuated by EZH2 knockdown (Fig. 2A–C). Next, we further confirmed the cell death phenotype of HCT116 cells by analyzing the expression of C-PARP, which is a key indicator of apoptosis. The increased levels of C-PARP observed under glucose deprivation conditions were attenuated by the knockdown of EZH2 (Fig. 2D). Similar experiments in SW480 cells also yielded the same results (Fig. 2E). To test the function of EZH2 enzymatic activity in glucose-deprivation-induced cell death, the EZH2 inhibitor GSK126 was used to interfere with its enzyme activity. The results showed that the cell apoptosis induced by glucose deprivation was significantly ameliorated in HCT116 and SW480 cells pretreated with GSK126 for 48 h (Fig. 2F, G). Correspondingly, the overexpression of EZH2 in CRC cells significantly enhanced the sensitivity of CRC cells to glucose deprivation, i.e., EZH2 overexpression promoted the cell death of glucose-sensitive cells and the cell cycle arrest of glucose-insensitive cells (Fig. 2H and Supplementary Fig. 2A). Consistently, EZH2 deficiency in HCT116 and SW480 cells attenuated the cell death caused by glucose deprivation, resulting in more live cells (Fig. 2I–K). Together, our data show that EZH2 deficiency alleviates the glucose dependence of CRC cells.

### EZH2 deletion promotes GLS expression and inhibits the glucose-deprivation-induced downregulation of GLS expression in glucose-sensitive CRC cells

**Fig. 3:**
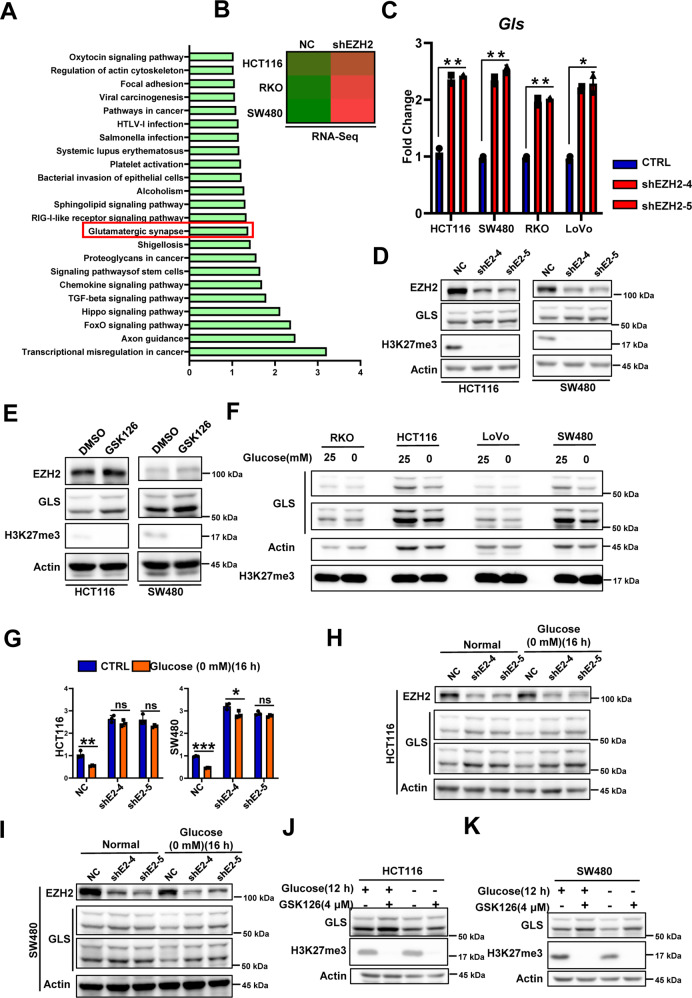
Absence of EZH2 function attenuates glucose-deprivation-induced downregulation of GLS. **A** KEGG pathway enrichment analysis showing pathways upregulated by EZH2 deficiency (https://david.ncifcrf.gov/). **B** RNA-Seq analysis of *GLS* expression in indicated CRC NC and shEZH2 cells. **C** RT-qPCR of *GLS* mRNA levels in indicated CRC NC and shEZH2 cells. **D, E** Immunoblotting for GLS expression in HCT116 or SW480 cells with (shE2-4/5) or without (NC) EZH2 knockdown (**D**), or treated with GSK126 (5 μM, 48 h) (**E**). Actin is used as a loading control. **F** Immunoblotting for GLS expression in indicated CRCs under glucose deprivation. **G** RT-qPCR of *GLS* mRNA levels in HCT116 or SW480 NC and shEZH2 cells with or without glucose deprivation treatment (0 mM, 16 h). **H, I** Immunoblotting for EZH2 and GLS expression in HCT116 (**H**) or SW480 (**I**) NC and shEZH2 cells with or without glucose deprivation treatment (0 mM, 16 h). **J, K** Immunoblotting for GLS and H3K27me3 expression in HCT116 (**J**) or SW480 (**K**) cells pretreated with or without GSK126 (4 μM, 48 h), followed by glucose deprivation treatment (0 mM, 12 h). The experiments in **D–F** and **H–K** were repeated twice independently with similar results. In **C**, **G**, data are mean ± s.d., *n* = 3 independent experiments; *P*-values were calculated using two-tailed unpaired Student’s *t*-test. *P < 0.05, **P < 0.01; ns, non-significant.

Next, we tried to analyze the relevant mechanisms by which EZH2 affects the glucose vulnerability of CRC cells. First, we investigated whether EZH2, a transcriptional regulator, could affect the expression of genes related to glucose metabolism. The RT-qPCR results and the subsequent RNA-Seq results showed that EZH2 deficiency could not regulate the expression of key enzymes of glucose metabolism (Supplementary Fig. 4A–C). The use of the glucose tracer 2-NBDG [18] also revealed that the knockdown of EZH2 did not affect glucose uptake by CRC cells (Supplementary Fig. 4D–G). To determine the molecular mechanism by which EZH2 regulates glucose vulnerability, we performed RNA-Seq analysis with two pairs of cell samples (HCT116^NC^ vs. HCT116^shEZH2^ and RKO^NC^ vs. RKO^shEZH2^). Intersection analysis of the upregulated genes (fold change >1.2) in the RNA-Seq data combined with the SW480 sequencing results in the GSE118593 dataset revealed that the expression of 224 genes was upregulated in the three cell lines due to EZH2 deletion (Supplementary Fig. 5A) [19]. KEGG pathway analysis of these 224 genes showed that FoxO signaling, Hippo signaling, TGF-β signaling, and other signaling pathways were significantly enriched (Fig. 3A). The first pathway that caught our attention was the Hippo signaling pathway, which negatively regulates the transcriptional coactivator Yap to affect cell proliferation and cell survival [20]. We hypothesized that the Hippo–Yap pathway might perform a function in glucose-deprivation-induced cell death similar to its function in ferroptosis; i.e., Yap activity is positively correlated with the cell death phenotype [21]. However, knocking down Yap expression significantly promoted the cell apoptosis induced by glucose deprivation (Supplementary Fig. 5B–D), and this finding could not explain our phenotype. A pathway named “glutamatergic synapse” then attracted our interest; in this pathway, the expression of glutaminase (GLS) appeared to be significantly increased (Fig. 3A, B). GLS is a key enzyme of glutamine metabolism that catalyzes the conversion of glutamine to glutamate [22]. Notably, the mRNA and protein levels of GLS were increased in EZH2-deficient and GSK126-treated cells cultured under normal conditions (Fig. 3C–E and Supplementary Fig. 2C). When the cells were cultured under glucose deprivation conditions, GLS expression was downregulated in glucose-sensitive CRC cells, and this effect was ameliorated by EZH2 inhibition (Fig. 3F–K and Supplementary Fig. 2D). However, although the mRNA level of *GLS* was significantly upregulated in RKO cells after EZH2 knockdown, the protein level of GLS remained unchanged, even when we prolonged the time of exposure to glucose deprivation conditions (Fig. 3C and Supplementary Fig. 2B). These phenotypes might be explained by the different requirements for glucose metabolism in glucose-sensitive and glucose-insensitive cells. In glucose-sensitive cells, glucose metabolism might play an essential role in redox homeostasis; therefore, glucose deprivation may ultimately result in ROS accumulation and cell death. However, in glucose-insensitive cells, glucose metabolism is needed to provide energy and intermediate metabolite supplies to maintain cancer cell proliferation. Collectively, our data suggest that EZH2 is a member of the network of cancer metabolism and that EZH2 deficiency upregulates GLS expression and blocks the glucose-deprivation-induced inhibition of GLS transcription in glucose-sensitive cells.

### GLS is the direct target gene of EZH2 under glucose deprivation conditions and its expression negatively correlates with the expression of *EZH2* in CRC tissues

**Fig. 4:**
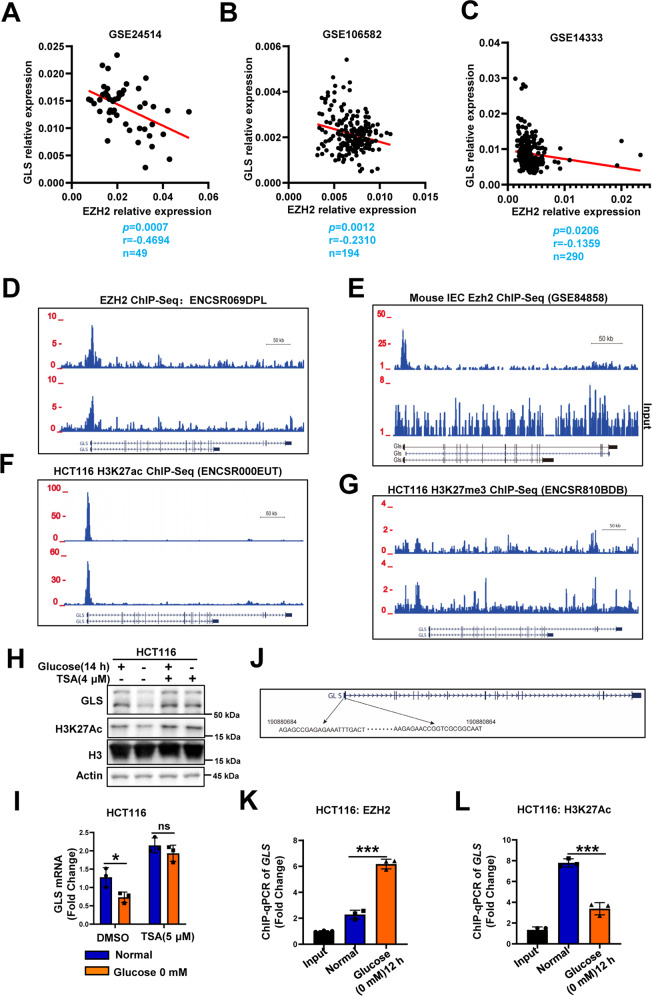
GLS is a direct target gene of EZH2 in colorectal cancer cells. **A–C** Scatter plots showing the correlation between *EZH2* and *GLS* mRNA levels in human colorectal cancer (GSE24514, GSE14333, and GSE106582). Statistical analysis was performed using a two-tailed Student’s *t*-test. r, Pearson’s correlation coefficient. **D** Schematic representation of the binding site of human EZH2 in *GLS* promoter as mapped in ENCODE database (ENCSR069DPL). **E** Schematic representation of the binding site of mouse EZH2 in *Gls* promoter of primary colon epithelial cells isolated from wild-type mice (GSE84858). **F**, **G** Schematic representation of the binding of H3K27ac (**F**) and H3K27me3 (**G**) on the *GLS* gene locus in HCT116 cells with the ENCODE project. **H**, **I** WB and RT-qPCR of *GLS* levels in CRCs pretreated with DMSO or HDAC inhibitor (TSA, 4 μM), followed by glucose deprivation treatment (0 mM, 16 h). **J** Location of ChIP-qPCR primers on GLS promoter. **K**, **L** ChIP-qPCR analysis of EZH2 and H3K27Ac on *GLS* promoter in HCT116 cells with or without glucose deprivation treatment (0 mM, 12 h). In **I**, **K**, and **L**, data are mean ± s.d., *n* = 3 independent experiments; *P*-values were calculated using two-tailed unpaired Student’s *t*-test. *P < 0.05, **P < 0.01, ***P < 0.001; ns, non-significant.

The data described above prompted us to further examine the clinical relevance of the EZH2-GLS relationship in human CRC. We examined the correlation between the expression of *EZH2* and *GLS* in the GSE24514, GSE14333, and GSE106582 datasets [23, 24]. The results showed that the *EZH2* levels were negatively correlated with those of *GLS* (Fig. 4A–C), indicating that *GLS* could be negatively regulated by EZH2 during CRC progression. ChIP interrogation of the *GLS* gene in ENCODE revealed the potential for the recruitment of EZH2 in human neural progenitor cells and hepatocytes (Fig. 4D) and more significant recruitment in mouse intestinal epithelial cells (Fig. 4E) [15, 25]. However, in HCT116 cells, the ENCODE results showed that H3K27Ac exhibited significant peaks in the *GLS* promoter, while either H3K27me3 or EZH2 did not in normal culture conditions (Fig. 4F, G and Supplementary Fig. 2E) [25]. This result made us think carefully and put forward the following hypothesis: Under normal conditions, GLS is continuously expressed, as shown by high H3K27Ac levels in its promoter. Under glucose deprivation conditions, H3K27Ac is eliminated, and EZH2 gains access to the *GLS* promoter, increasing the methylation of H2K27 and thereby inhibiting the transcription of *GLS*. Since the *GLS* promoter had the potential to be directly bound by EZH2 and the decrease in GLS expression by glucose deprivation was attenuated by EZH2 silencing (Fig. 4D–G and Fig. 3), we sought to investigate whether EZH2 was more inclined to bind to the *GLS* promoter under glucose deprivation conditions. To test this hypothesis, we first used western blotting to check H3K27Ac and found it could be significantly downregulated under glucose deprivation treatment (Fig. 4H). Then we performed ChIP analysis in HCT116 cells cultured under normal conditions or glucose deprivation conditions and found that EZH2 could directly bind to the *GLS* promoter and that the recruitment of EZH2 to the *GLS* promoter was significantly increased under glucose deprivation conditions (Fig. 4K). As H3K27Ac and H3K27me are antagonistic, we also proved that the modification of H3K27Ac in the *GLS* promoter is decreased (Fig. 4L). Further, we pretreated HCT116 cells with the HDAC inhibitor trichostatin A (TSA). The results showed that the downregulation of GLS expression and the reduction in H3K27Ac modification in the *GLS* promoter induced by glucose deprivation were inhibited by TSA treatment (Fig. 4H, I).

**Fig. 5:**
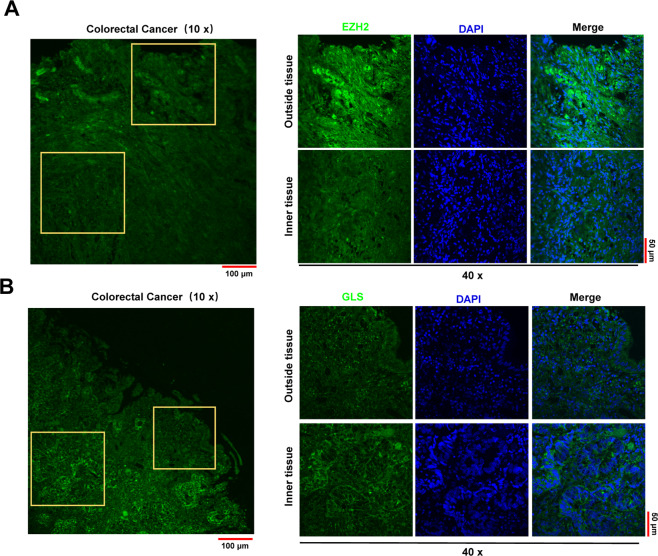
GLS expression has a negative spatial correlation with EZH2 in colorectal cancer tissues. **A, B** Immunofluorescence of EZH2 (A) and GLS (B) in human colorectal cancer samples. DAPI is used to indicate the nucleus.

Solid tumors exhibit significant heterogeneity in terms of vasculature perfusion. Cells located in the outside layer of the tumor tissue are highly perfused, while the innermost cells are poorly perfused, residing in nutrient-poor environments. Therefore, to further confirm the involvement of the EZH2-GLS axis in the adaptation to glucose deprivation, we performed immunofluorescence staining for EZH2 and GLS in larger colon cancer tissues. The results showed that EZH2 was highly expressed in the outer layers of tumor tissues, while GLS was highly expressed in the inner layers (Fig. 5A, B). These data indicated that the expression levels of EZH2 and GLS changed dramatically due to different locations in tumor tissues, which explained why the RNA sequencing data did not show a good correlation between EZH2 and GLS expression in CRC (Fig. 4A–C). Taken together, these results suggest that EZH2 can directly regulate the expression of GLS in CRC and that this correlation is related to the nutritional conditions in the microenvironment.

### GLS is involved in the resistance of EZH2-deficient cells to glucose deprivation

**Fig. 6:**
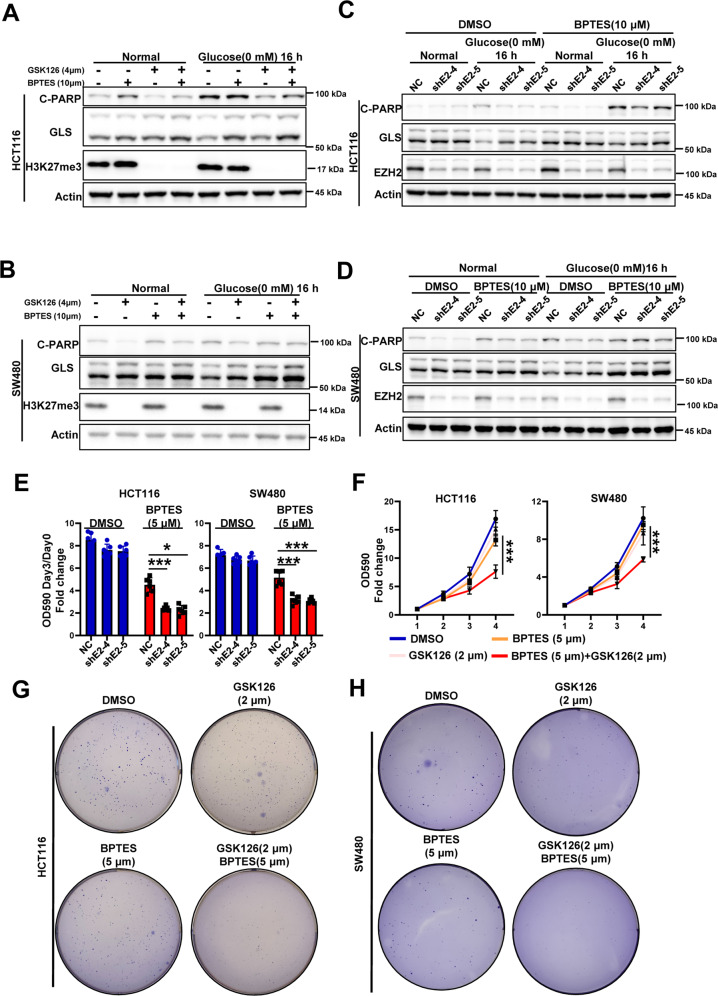
GLS is involved in the resistance of EZH2-deficient cells to glucose deprivation. **A, B** Immunoblotting for cleaved-PARP and GLS expression in HCT116 (**A**) or SW480 (**B**) cells pretreated with or without GSK126 (4 μM, 48 h) or/and BPTES (10 μM, 48 h), followed by glucose deprivation treatment (0 mM, 16 h). Actin is used as a loading control. **C, D** Immunoblotting for cleaved-PARP and GLS expression in HCT116 NC and shEZH2 cells (**C**) or SW480 NC and shEZH2 cells (**D**) cells pretreated with DMSO or BPTES (10 μM, 48 h), followed by glucose deprivation treatment (0 mM, 16 h). **E** Three-day growth of HCT116 NC and shEZH2 cells or SW480 NC and shEZH2 cells in presence or not of 5 μM BPTES. **F** Growth curves of HCT116 or SW480 cells in presence or not of 2 μM GSK126 or/and 5 μM BPTES. **G, H** Colony formation of HCT116 (**G**) or SW480 (**H**) cells in presence or not of 2 μM GSK126 or/and 5 μM BPTES. The experiments in **A–D** and **G**, **H** were repeated twice independently with similar results. In **E**, **F**, data are mean ± s.d., *n* = 3 independent experiments; *P*-values were calculated using two-tailed unpaired Student’s *t*-test or two-way ANOVA test. *P < 0.05, **P < 0.01, ***P < 0.001.

The results described above showed that EZH2 knockdown enhanced the resistance of CRC cells to glucose deprivation and that *GLS* expression was elevated in EZH2-knockdown cells. Thus, we next investigated whether the resistance of EZH2-deficient cells to glucose deprivation was due to the upregulation of GLS expression. As a key enzyme in glutamine metabolism, GLS has always been an attractive anticancer therapeutic target. A variety of inhibitors that target GLS have entered the clinical trial stage [26, 27]. Here, bis-2-(5-phenylacetamido-1,2,4-thiadiazol-2-yl) ethyl sulfide (BPTES) was chosen to inhibit the activity of GLS and determine whether the resistance could be rescued. By pretreating EZH2-deficient CRC cells with BPTES, we found that BPTES treatment significantly reversed the decreased cell death phenotype caused by EZH2 deletion or inhibition (Fig. 6A–D). Furthermore, BPTES and EZH2 silencing/inhibition synergistically inhibited the proliferation rate of CRC cells (Fig. 6E, F). Colony formation assays further confirmed the synergistic effect of the EZH2 inhibitor and GLS inhibitor (Fig. 5G, H and Supplementary Fig. 6E). Then, we wondered whether other molecules that modulate GLS activity also exhibit a synergistic effect with EZH2 inhibitors; one such molecule is mTOR (Supplementary Fig. 6 A), which promotes GLS activity and glutamine metabolism [28, 29]. The results showed that the combined treatment of rapamycin and GSK126 significantly inhibited cancer cell proliferation and colony formation (Supplementary Fig. 6B–D). Collectively, these data suggest that targeting glutamine metabolism has the potential to enhance the efficacy of EZH2 inhibitors and reduce CRC tolerance.

### The EZH2-GLS axis regulates glucose-deprivation-induced cell death by modulating intracellular GSH levels

**Fig. 7:**
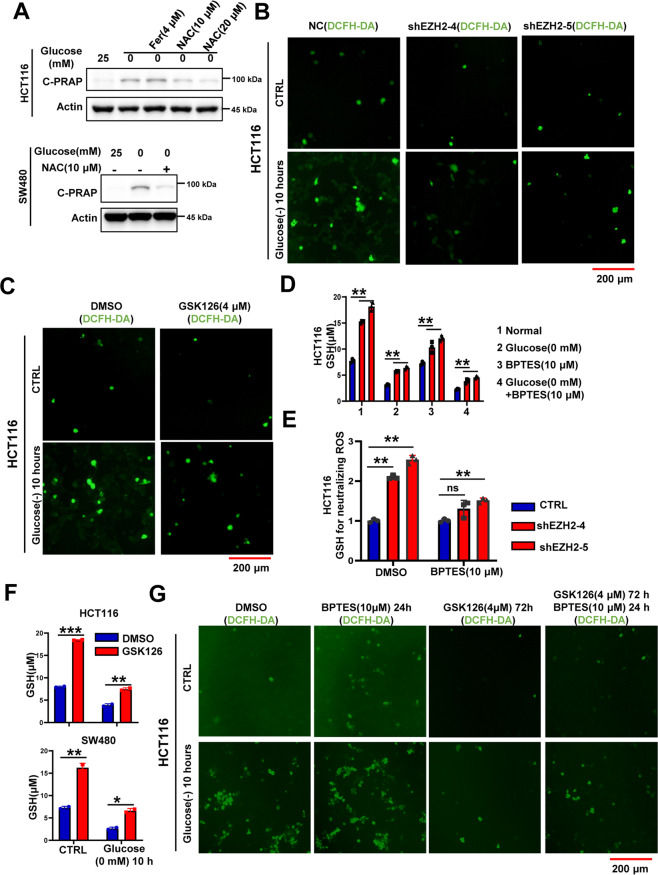
The EZH2-GLS axis regulates glucose-deprivation-induced cell death by modulating intracellular GSH levels. **A** Immunoblotting for cleaved-PARP in HCT116 or SW480 cells cultured with or without glucose deprivation (0 mM, 16 h) in presence or not of 4 μM Fer or 10 μM/20 μM NAC (*n* = 2 independent experiments). **B** DCFH staining of HCT116 NC and shEZH2 cells under normal condition or glucose deprivation (0 mM, 10 h). **C** DCFH staining of HCT116 cells pretreated with DMSO or GSK126 (4 μM, 48 h), followed by glucose deprivation treatment (0 mM, 10 h). **D** Intracellular GSH content of HCT116 cells pretreated with DMSO or BPTES (10 μM, 48 h), followed by glucose deprivation treatment (0 mM, 10 h). **E** Intracellular GSH content for neutralizing ROS in HCT116 NC and shEZH2 cells pretreated with DMSO or BPTES (10 μM, 48 h), followed by glucose deprivation treatment (0 mM, 10 h). **F** Intracellular GSH content of HCT116 or SW480 cells pretreated with DMSO or GSK126 (4 μM, 48 h), followed by glucose deprivation treatment (0 mM, 10 h). **G** DCFH staining of HCT116 cells pretreated with or without BPTES (10 μM, 24 h) or/and GSK126 (4 μM, 72 h), followed by glucose deprivation treatment (0 mM, 10 h). The experiments in **A**–**C** and **G** were repeated twice independently with similar results. In **D**–**F**, data are mean ± s.d., *n* = 3 independent experiments; *P*-values were calculated using two-tailed unpaired Student’s *t*-test. *P < 0.05, **P < 0.01, ***P < 0.001; ns, non-significant; scale bars, 200 μm.

As a key component of redox balance, ROS plays an important role in the cell death induced by glucose deprivation [1, 30]. In this study, we first used the ROS scavenger NAC to treat glucose-deprived cells and found that glucose-deprivation-induced cell death was significantly attenuated by NAC (Fig. 7A); these results indicated that glucose-deprivation-induced cell death in CRC was mediated by ROS. Furthermore, EZH2 knockdown or inhibition significantly reduced intracellular ROS accumulation under glucose deprivation conditions (Fig. 7B, C), which was consistent with the cell death phenotype (Fig. 2). During glutamine metabolism, GLS converts intracellular glutamine into glutamate, which is used to exchange extracellular cystine through xCT, thereby promoting GSH synthesis and preventing excessive ROS accumulation [30, 31]. Our results showed that EZH2 knockdown or inhibition significantly increased the intracellular GSH content and that BPTES treatment attenuated the increase in GSH content and the decrease in ROS levels caused by EZH2 deletion under glucose deprivation conditions (Fig. 7D–G). Collectively, these data suggest that the EZH2-GLS axis regulates glucose vulnerability by modulating the intracellular GSH and ROS levels.

## Discussion

Metabolic reprogramming is a hallmark of malignantly transformed cells, making them highly dependent on some nutrients such as glucose [32]. However, during cancer progression or clinical drug intervention, local nutrient deficiency inevitably occurs in cancer tissues. In these cases, how cancer cells adapt to nutrient deprivation to survive and whether epigenetic plasticity is involved in this process are meaningful questions worth exploring in depth. Here through conditional screening of an epigenetic sgRNA library, we found that EZH2 played an important role in the tolerance of colorectal cancer cells to glucose deprivation. In cancer cells facing glucose limitation, the expression of EZH2 was downregulated and EZH2 deficiency attenuated glucose-deprivation-induced cell death and cell cycle arrest. In solid tumors, EZH2 generally exists as an oncogene, mainly through promoting proliferation, anti-apoptosis, and other biological processes [11]. Although our previous studies demonstrated the cancer-promoting role of EZH2 in the occurrence and progression of colorectal cancer in mice models, this study about glucose deprivation suggested that loss-of-EZH2 can also play a cancer promoting role in certain conditions (Supplementary Fig. 7). The contradiction of these two different roles enlightened us that the occurrence and progression of cancer are different, and heterogeneity of cancer tissues should be taken into consideration under specific conditions. Further, we showed that EZH2 was indeed highly expressed in cancer tissues, but the prognosis of patients was not affected by the expression of EZH2, which was consistent with the conclusion of the previous study [33].

Targeting EZH2 to treat cancer is a hot spot in current clinical research and some inhibitors have been approved by FDA to treat cancer [34]. However, the resistance of cancer cells to EZH2 inhibitors and the underlying mechanisms still need to be explored. In this study, we showed the tumor-suppressive function of EZH2 was mediated by inhibiting the expression of *GLS*, which played a vital role in cancer metabolic reprogramming. These results might be an explanation for the resistance of cancer cells to EZH2 inhibitors. Mechanistically, we found the metabolism-related gene *GLS* could be directly targeted by EZH2. Although EZH2 expression was downregulated under glucose deprivation, the binding of EZH2 on the promoter region of *GLS* was significantly enhanced, leading to the downregulation of GLS expression. Meanwhile, the loss of EZH2 function resulted in the upregulation of GLS expression, and GLS expression was no longer reduced by glucose deprivation. As a key enzyme of glutamine metabolism, GLS functions as a cancer driver and can also be a therapeutic target in cancer. Under glucose deprivation ROS was accumulated in cancer cells due to the rapidly declining GSH level and shutting down the pentose phosphate pathway. Then the importance of increased GLS induced by EZH2 deficiency was amplified, as GLS converts glutamine to glutamate, which can be used to exchange cystine from extracellular regions and facilitate the synthesis of glutathione to clear ROS. Furthermore, we also proved that the inhibitors that target GLS activity or upstream regulators of glutamine metabolism like mTOR showed synergistic effects with EZH2 inhibitors.

This work and previous studies suggested that the heterogeneity and the plasticity of cancer cells make them respond differently to the same treatment and the existence of this phenomenon to some extent explains the drug resistance to single-target treatments [35–37]. In the context of precision medicine, a better understanding of the heterogeneity of cancer tissues will facilitate the collaborative medication of cancer [38]. Of course, there are still some unresolved issues in our study. For example, cell apoptosis induced by BPTES is also weakened by the loss of EZH2, which HDAC molecule is responsible for the deacetylation of the H3K27ac in the *GLS* promoter. Taken together, our study shows the tumor-suppressive function of EZH2 under nutrient-deficient conditions, which partially explains why the clinical expression of EZH2 does not affect the prognosis of CRC patients and reveals a possible mechanism of the resistance of cancer cells to EZH2 inhibitors from the perspective of metabolism.

## Materials and methods

### Cell culture and reagents

All cancer cell lines were obtained from the American Type Culture Collection (ATCC, Manassas, VA, USA). All cell lines were free of mycoplasma contamination (tested by the PCR). Cells were cultured in Dulbecco’s modified Eagle medium (DMEM) (Thermo Fisher Scientific, Waltham, MA, USA) with 10% fetal bovine serum (Biological Industries, Kibbutz Beit Haemek, Israel) at 37 °C with 5% CO_2_. For glucose deprivation experiments, cells were cultured in 0 mM Glucose DMEM with 10% FBS for indicated time and cells were washed by PBS before medium changing. DMEM without glucose was obtained from Thermo Fisher Scientific (11966-025). GSK126 (HY-13470), BPTES (HY-12683), TSA (HY-15144) were got from MedChemExpress (MCE) (NJ, USA). DCFH-DA Reactive Oxygen Species Assay Kit, GSH, and GSSG Assay Kit were purchased from Beyotime Institute of Biotechnology (Shanghai, China). NAC (A7250) was purchased from Sigma-Aldrich (St. Louis, MO, USA).

### Stable cell lines generation

EZH2 short hairpin RNA (shRNA) were constructed with pLKO.1 system and lentiviral plasmid over expressing EZH2 was purchased firm Hanbio (Shanghai, China). Cell lines with specific gene silence or overexpression were generated as previously described. In brief, HEK293T cells were transfected either with pLKO.1 or pLV plasmid, together with Δ8.9 and VSVg third-generation lentiviral packaging system using Lipofectamine 2000 reagent (Thermo Fisher Scientific) according to the manufacturer’s instructions. After transfection, lentivirus particles in the medium were collected every 24 h, and after three collections the medium was filtered, then the target cell lines were infected. At 48 h after infection, 2 μg/ml puromycin was added to obtain stable cell lines with successful transduction. The sequences of shRNAs used in this study are listed in Supplementary Table 4.

### Western blotting

Western blotting was conducted as previously described [15, 39]. The primary antibodies and concentrations used for Western blotting were following: EZH2 (1:1000, 5246 S, Cell Signaling Technology, Danvers, MA, USA), H3K27me3 (1:1,000, 9733 S, Cell Signaling Technology), C-PARP (1:2,000, 5625 S, Cell Signaling Technology), GLS (1:1,000, 56750 T, Cell Signaling Technology), Actin (1:10,000, AP0060, Bio-world, Nanjing, China).

### Reactive oxygen species (ROS) and GSH assay

For ROS measurement, colorectal cancer cells were seeded in a 12-well plate and treated with corresponding conditions. After the treatment, the medium in the well was transferred to the corresponding EP tube, and then 400 ul medium was added back to the original well. DCFH-DA was added to the wells at a ratio of 1:1,000, mixed and incubated at 37 °C for 15 min. Then the dye solution was removed, the medium in the corresponding EP tubes was added back to the corresponding wells, and observation and photographing were performed through a fluorescence microscope (Olympus IX70). To detect GSH content, cells were treated as indicated and prepared for measurement of glutathione using the GSH and GSSG Assay kit according to the manufacturer’s instructions. The concentration of GSH was calculated using a standard curve.

### RNA-Seq and ChIP-qPCR analysis

Total RNA was subjected to MGI2000 performed by BGI Tech Solutions Co., Ltd. Transcriptomic reads were mapped to reference genome (mm10) using Bowtie software, and gene expression levels were quantified using the RSEM software package. The list of significantly affected genes was obtained by setting a false discovery rate (FDR) threshold of 0.001, *P*-value < 0.0,1 and fold changes greater than 1.2 or log2Fc < −0.75. Manual curation of GOs was performed using KEGG or Enrichr and visualized in the form of a heatmap using −Log10(*P*-value). Gene expression datasets were deposited into the GEO database (GSE159351 and GSE159352). The ChIP assays were performed using the Magnetic ChIP kit (Millipore). The procedure was as described in the kit provided by the manufacturer. Briefly, isolated HCT116 cells were fixed by 1% formaldehyde, fragmented by sonication. EZH2 (Cell Signaling Technology, 5246 S) and H3K27Ac (Cell Signaling Technology, 8173 S) were then used for immunoprecipitation. After washing and reverse-crosslinking, the precipitated DNA was amplified by primers and quantified by the qPCR. Primer sequences can be found in Supplementary Table 4.

### Cell number assays and colony formation assay

The cells at 100% confluency were seeded in 12-well plate 1 day before treatment to measure living cells. After treatment as indicated, cells were trypsinized and collected in a new EP tube and stained with 0.4% Trypan Blue Solution. The number and proportion of dead and living cells were calculated using CountessII FL. To measure cell viability, cells were seeded in a 96-well plate 1 day before treatment. Following the treatment with the appropriate drugs as indicated, cells were exposed to 10 μl resazurin sodium salt. After incubation for 2 h, the plate was analyzed using the Paradigm Detection Platform (Beckman, CA, USA). Colony formation assay was done as previously described [39].

### Specimens

Paraffin-embedded colorectal cancer tissues were collected from patients with CRC who had undergone routine surgery at the Nanfang Hospital, Southern Medical University (Guangzhou, China) and volunteered to provide samples for research.

### Immunofluorescence

Immunofluorescence analysis of EZH2 and GLS was performed on paraffin-embedded tissue from colorectal cancer patients. To expose target proteins, antigen retrieval was performed using 10 mM sodium citrate (pH 6.0), microwaved for 8–15 min. Following antigen retrieval, tissues were blocked in 3% H_2_O_2_-methanol for 15 min at room temperature, washed with ddH_2_O and PBS, and then probed with EZH2 antibody (5246 S) or GLS antibody (56750 T) diluted in 3% BSA-PBS at a dilution of 1:40 for 12 h at 4 °C in a humidified chamber. Tissues were washed extensively in PBST and incubated with an FITC-conjugated secondary antibody (Thermo Fisher Scientific) for 2 h. The nuclei were labeled with DAPI (Thermo Fisher Scientific), and fluorescence was monitored using a confocal microscope (Olympus).

### Survival analysis and gene expression correlation analysis

The READ and COAD datasets of the TCGA database were used for expression and survival analysis [40]. Expression profiles of GSE24514, GSE14333, and GSE106582 were downloaded from GEO [41–43]. Statistical computations were performed using GraphPad Prism8. Statistical significance was assessed by One-way ANOVA; the data were considered not significant for p > 0.05. Correlation values between two genes were determined by iterating through each probe associated with gene *EZH2* and comparing it to probe associated with gene *GLS*. Statistical significance was determined by the Pearson correlation coefficients test.

### Mice experiments

All mice were maintained in a specific-pathogen-free (SPF) facility, and experimental procedures were approved by the institutional biomedical research ethics committee of the Shanghai Institutes for Biological Sciences or Institute of Zoology, Chinese Academy of Sciences and Animal Ethics Committee of Southern Medical University. EZH2^IEC−/−^ mice were generated by crossing EZH2 Flox mice [44] with Villin-Cre [45]. Descendants of APC^min/+^ mouse [46] and EZH2 ^IEC−/−^ are used to research spontaneous colon cancer. In AOM/DSS experiments, mice were first treated with a single dose of AOM (Sigma), and then 1% DSS (molecular weight, 36–50 kDa; MP Biomedicals, 160110) was given in the drinking water for 4 days, followed by 2 weeks of regular drinking water. The DSS treatment was repeated for two additional cycles, and mice were sacrificed 10 weeks after AOM injection. All mice were maintained on a C57BL/6 background.

### Statistical analysis

All experiments were independently performed three times unless otherwise stated. Data are presented as mean ± standard deviation (s.d.). Pearson correlation coefficients were used to evaluate the relationships between EZH2 and GLS expression. Statistical significance was determined by Student’s *t*-test, One-way ANOVA, Log-rank test, or Fisher’s exact test. For all statistical tests, the 0.05 level of confidence (two-sided) was accepted for statistical significance.

## Supplementary information

Supplementary

Supplementary Table 1

Supplementary Table 2

Supplementary Table 3

Supplementary Table 4
