# Supplementary material for: Tumor-suppressive function of EZH2 is through inhibiting glutaminase
Source: Cell Death Dis. 2021 Oct 20;12(11):975. doi: 10.1038/s41419-021-04212-7 (PMC8528894; doi:10.1038/s41419-021-04212-7)

Supplementary Figure. 1 The effects of glucose deprivation on the expression of PRC2 complex genes.

A

sgRNA	Target	Group S	Group R	R/S
S_1244	EZH2	159	294	1.849057
S_1245	EZH2	4170	6530	1.565947
S_1242	EZH2	722	1243	1.721607
S_1243	EZH2	127	257	2.023622
S_1311	SUZ12	12130	18370	1.514427
S_1313	SUZ12	6087	8500	1.396419
S_1310	SUZ12	573	758	1.322862
S_1312	SUZ12	3437	2554	0.74309
S_2379	EED	14387	13406	0.931813
S_2380	EED	436	306	0.701835
S_2381	EED	834	576	0.690647
S_1226	PHF19	755	1409	1.866225
S_1225	PHF19	1550	2111	1.361935
S_1227	PHF19	2232	2024	0.90681
S_1228	PHF19	9114	6288	0.689928

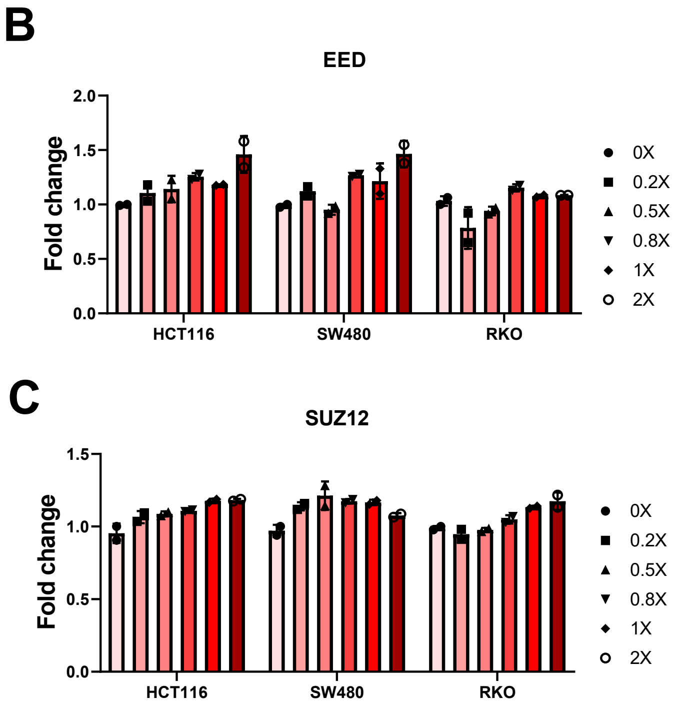

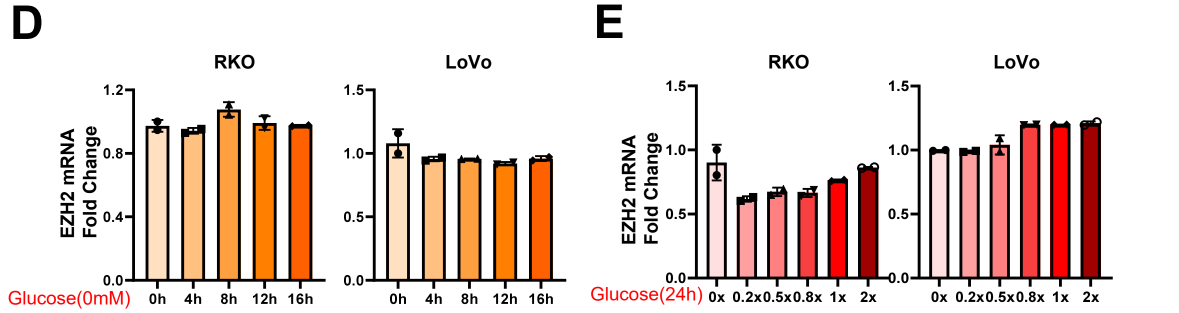

A. Table illustrating sgRNA targeting PRC2 complex genes enriched in group R (Resistance) and S (Sensitive). **B-C.** RT-qPCR of EED (B) or SUZ12 (C) levels in HCT116, SW480, and RKO cells treated with different doses of glucose as indicated. **D-E.** RT-qPCR of EZH2 levels in RKO and LoVo cells under glucose deprivation for indicated time (D) or treated with indicated doses of glucose for 24 h (E). 1×glucose, 25 mM.

Supplementary Figure. 2 EZH2 deletion promotes GLS expression and inhibits glucose-induced downregulation of GLS in HCT116 cells, but not in RKO cells.

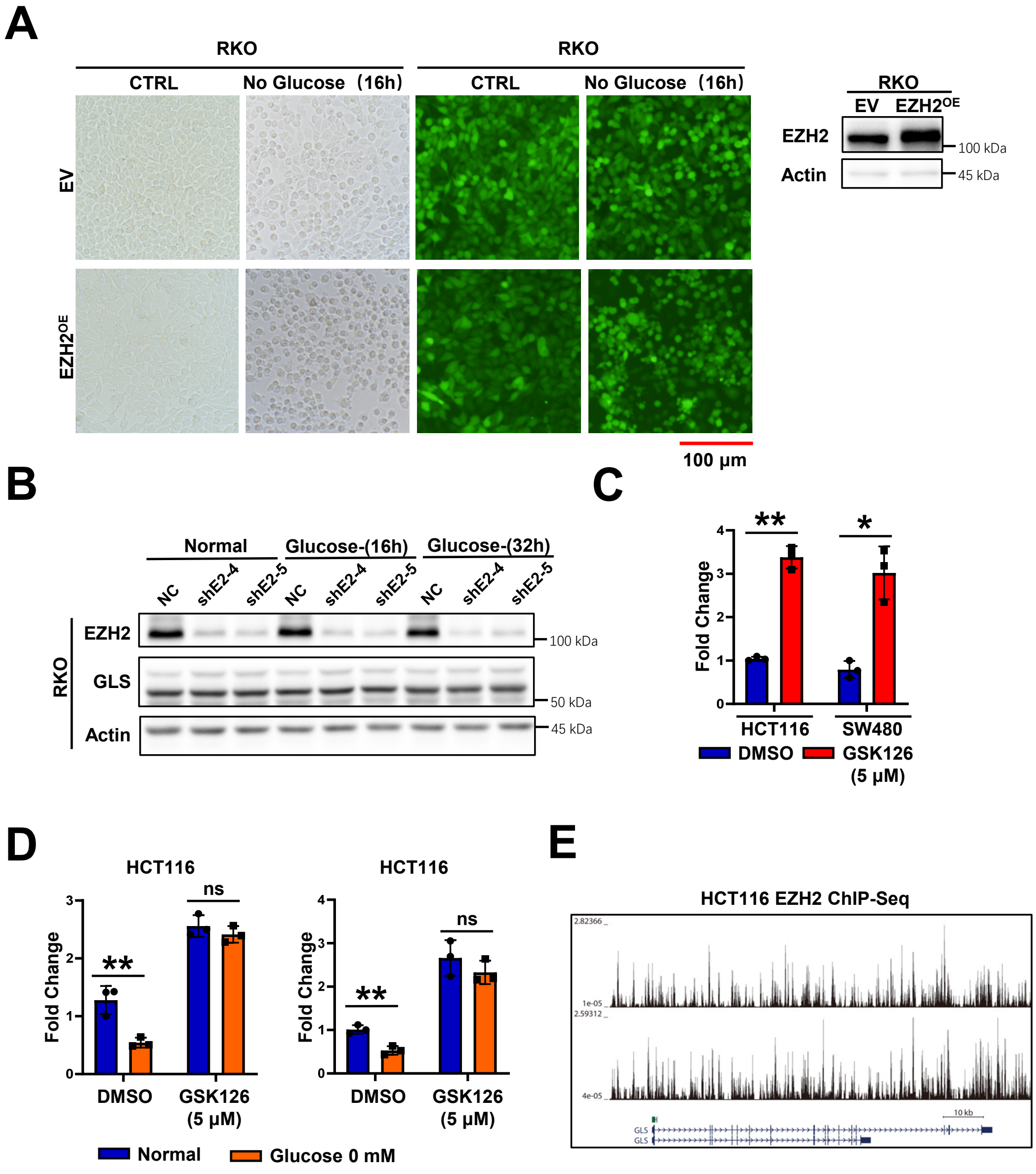

A. Images (left) and immunoblotting for EZH2 expression (right) in RKO EV and EZH2^{OE} cells under glucose deprivation for 16 h. **B.** Immunoblotting for EZH2 and GLS expression in RKO NC and shEZH2 cells under glucose deprivation for indicated time. Actin is used as a loading control. **C.** qPCR of GLS levels in HCT116 and SW480 cells treated with or without 5 μM GSK126. **D.** qPCR of GLS levels in HCT116 pretreated with or without 5 μM GSK126, followed by glucose deprivation. **E.** Schematic representation the binding of EZH2 on the *GLS* gene locus in HCT116 cells with the ENCODE project.

Supplementary Figure. 3 EZH2 promotes tumorigenesis in the colon.

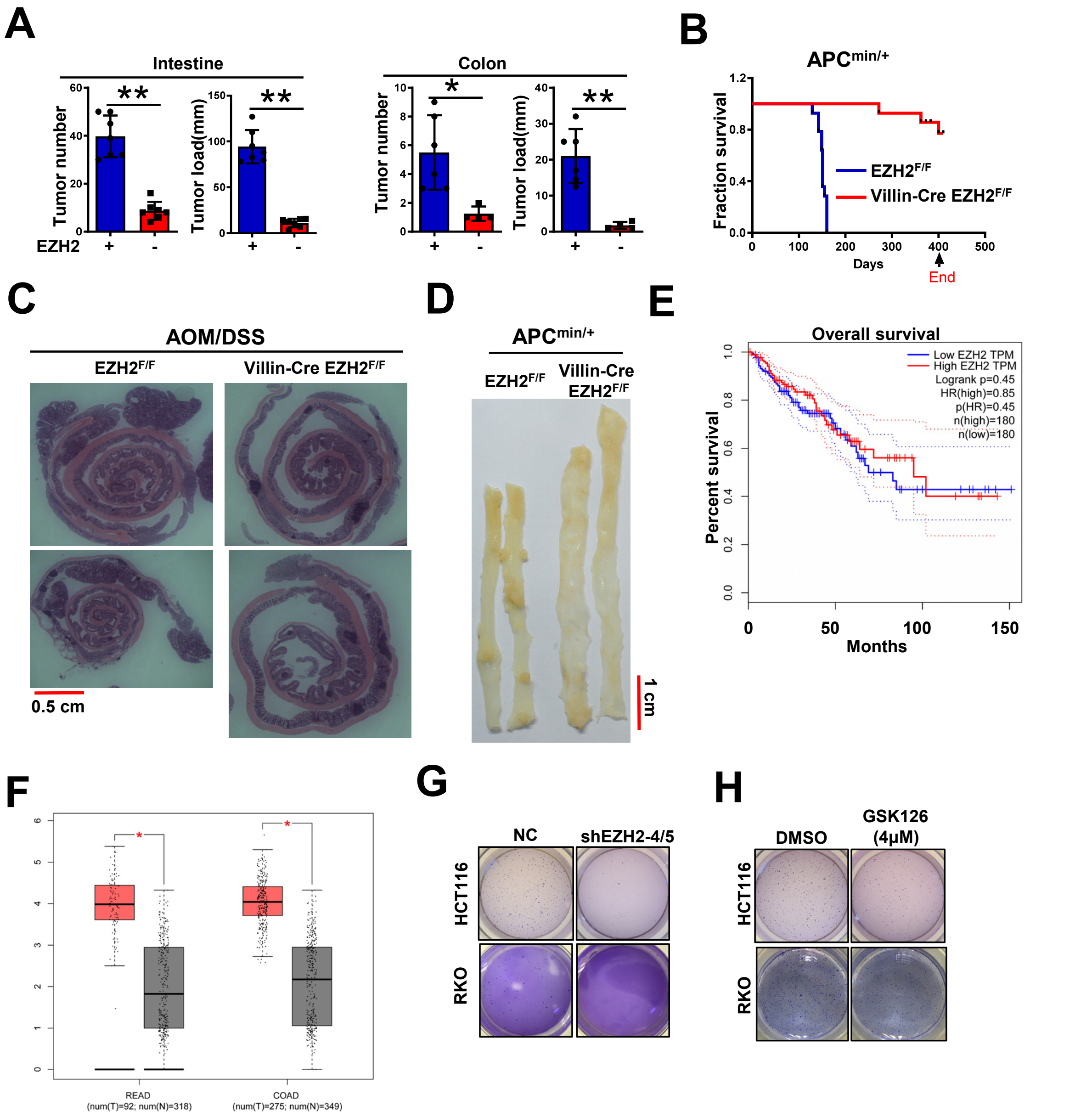

A. Tumor number and tumor load in intestine (left) and colon (right) from $APC^{min/+}$; $EZH2^{F/F}$ and $APC^{min/+}$; Villin-Cre; $EZH2^{F/F}$ mice that were 4 months old. **B.** Survival of $APC^{min/+}EZH2^{F/F}$ (n=10) and $APC^{min/+}$; Villin-Cre; $EZH2^{F/F}$ (n=10) mice. **C.** H&E staining of tumor counts in intestinal sections from AOM/DSS treated $EZH2^{F/F}$ and Villin-Cre; $EZH2^{F/F}$ mice . **D.** Images of tumor counts in the colons of $APC^{min/+}$; $EZH2^{F/F}$ and $APC^{min/+}$; Villin-Cre; $EZH2^{F/F}$ mice. **E.** Kaplan–Meier plots of colorectal cancer patients stratified by EZH2 expression. **F.** Boxplot of EZH2 expression levels in healthy controls (grey) and colorectal cancer patients (Red), TCGA. **G-H.** Colony formation of HCT116 or RKO NC and shEZH2 cells (g) or HCT116 or RKO cells treated with or without 4 μM GSK126 (h).

Supplementary Figure. 4 The effects of EZH2 knockdown on the expression of glycolysis related genes and glucose uptake

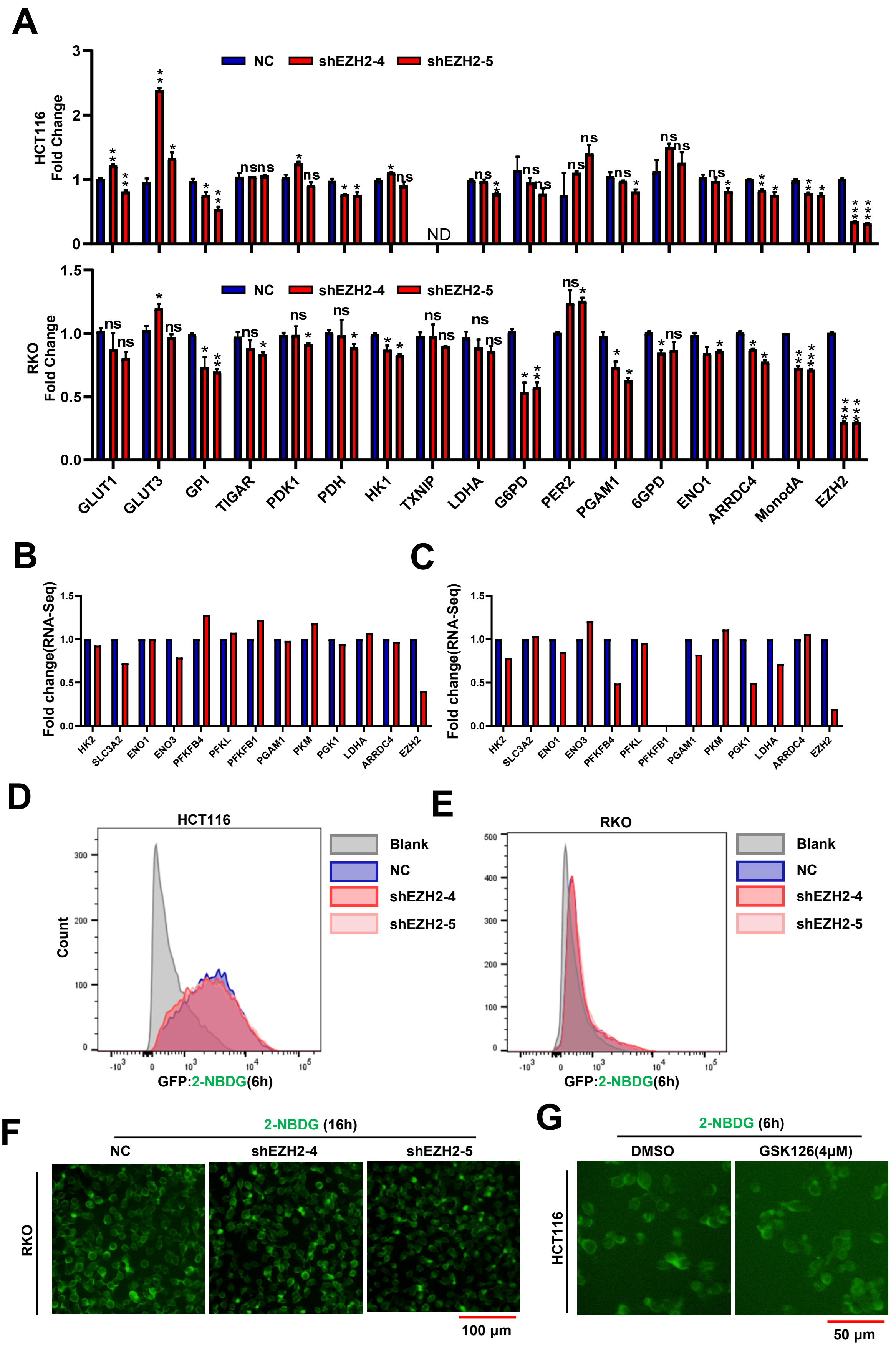

A. qPCR of glycolysis related genes and EZH2 levels in HCT116 (up) or RKO (down) NC and shEZH2 cells. **B-C.** RNA-Seq analysis of glycolysis related genes and EZH2 levels in HCT116 (b) or RKO (c) NC and shEZH2 cells. **D-E.** FACS analysis of glucose uptake in HCT116 (d) or RKO (e) NC and shEZH2 cells treated with 2-NBDG for 6 h. **F-G.** Images of glucose uptake using 2-NBDG for indicated time in RKO NC and shEZH2 cells (f) or HCT116 cells pretreated with or without 4 μM GSK126 (g).

Supplementary Figure. 5 EZH2 regulation of glucose vulnerability is not mediated through Hippo-Yap pathway.

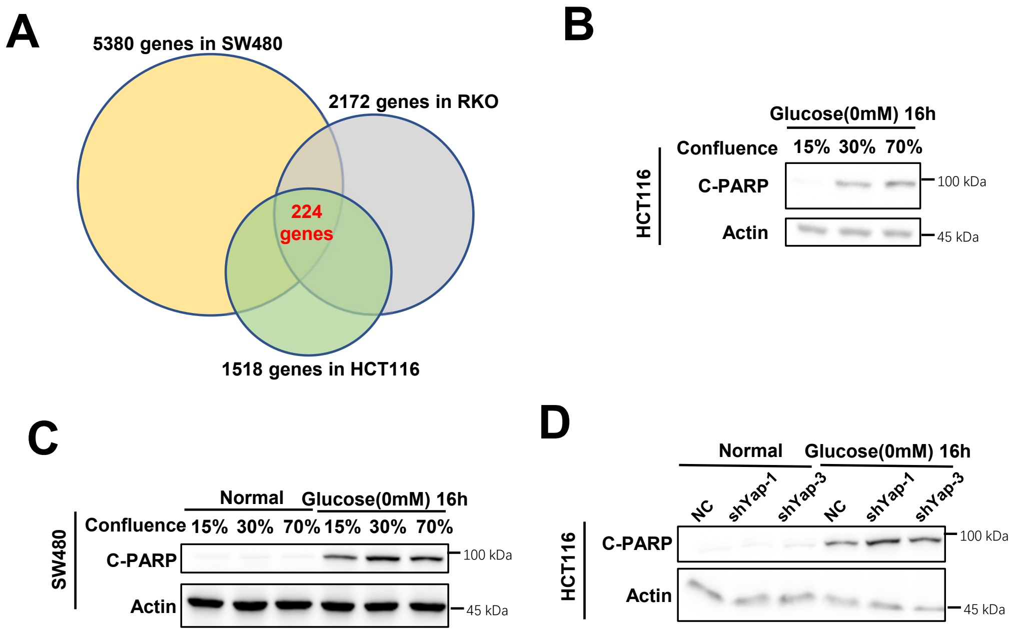

A. Venn diagram showing the overlap between genes upregulated by EZH2 knockdown in HCT116 and RKO cells, and genes upregulated by EZH2 knockdown in SW480 sequencing dataset GSE118593. **B-C.** Immunoblotting for cleaved-PARP expression in HCT116 (b) or SW480 (c) cells seeded at the indicated density under glucose deprivation for 16 h. **D.** Immunoblotting for cleaved-PARP expression in HCT116 NC and shYap cells under glucose deprivation for 16 h. Actin is used as a loading control.

Supplementary Figure. 6 EZH2 inhibitor and mTOR inhibitor synergistically inhibit CRC cell growth.

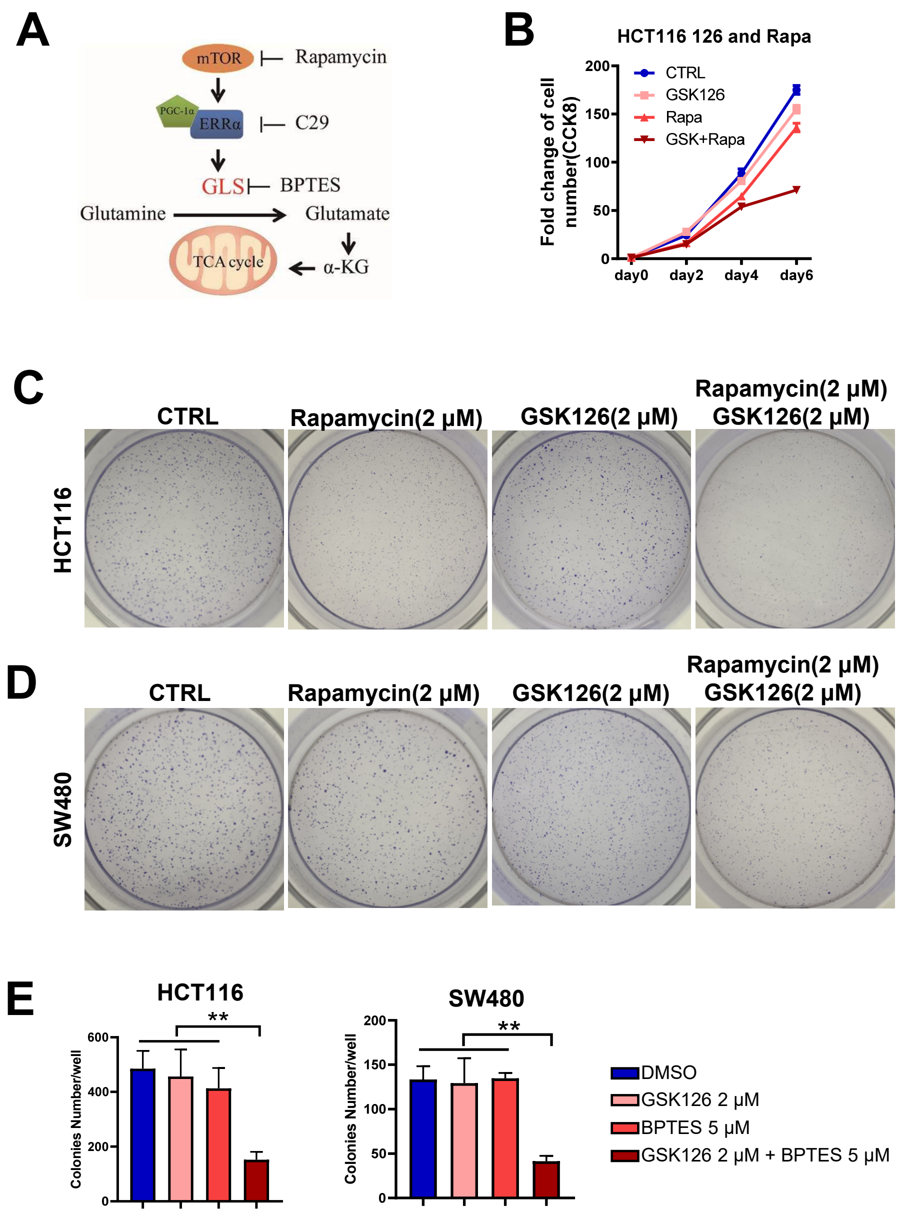

A. Schematic diagram showing mTOR inhibitor rapamycin could inhibit GLS activity. **B.** Growth curves of HCT116 cells in presence of 2 μM GSK126 or/and 2 μM rapamycin. **C-D.** Colony formation of HCT116 (d) or SW480 (e) cells in presence of 2 μM GSK126 or/and 2 μM rapamycin. **E.** Synergistic treatment of EZH2 inhibitor and GLS inhibitor significantly reduce the colony formation of CRC cells in soft agar.

Supplementary Figure. 7 The working model depicting how EZH2 regulates GLS-GSH and glucose-deprivation-induced cell death.

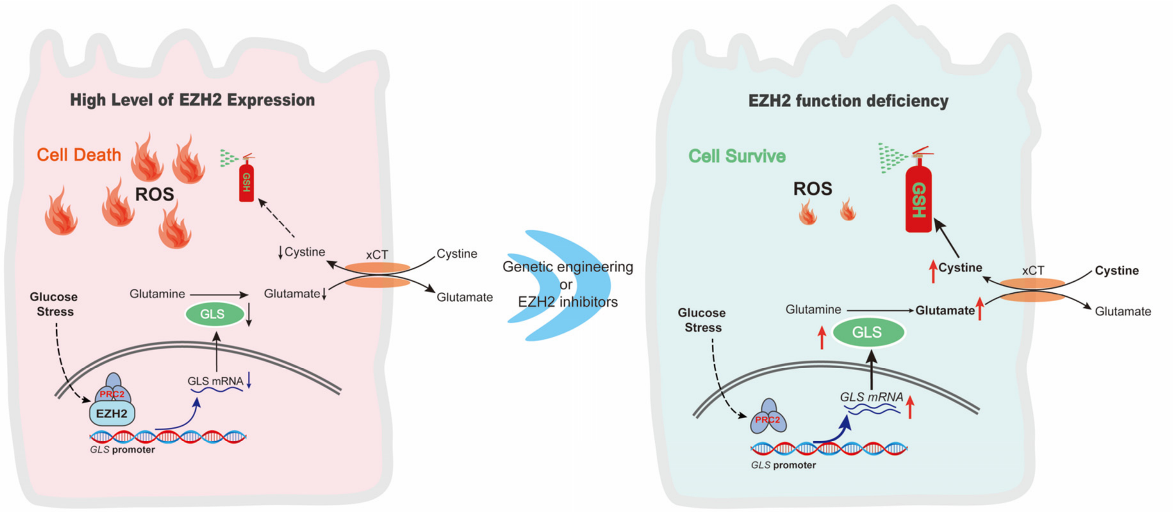

EZH2 deficiency causes high GLS expression, thereby facilitating GSH synthesis and attenuating glucose-deprivation-induced ROS accumulation and cell death.

Figure 1A

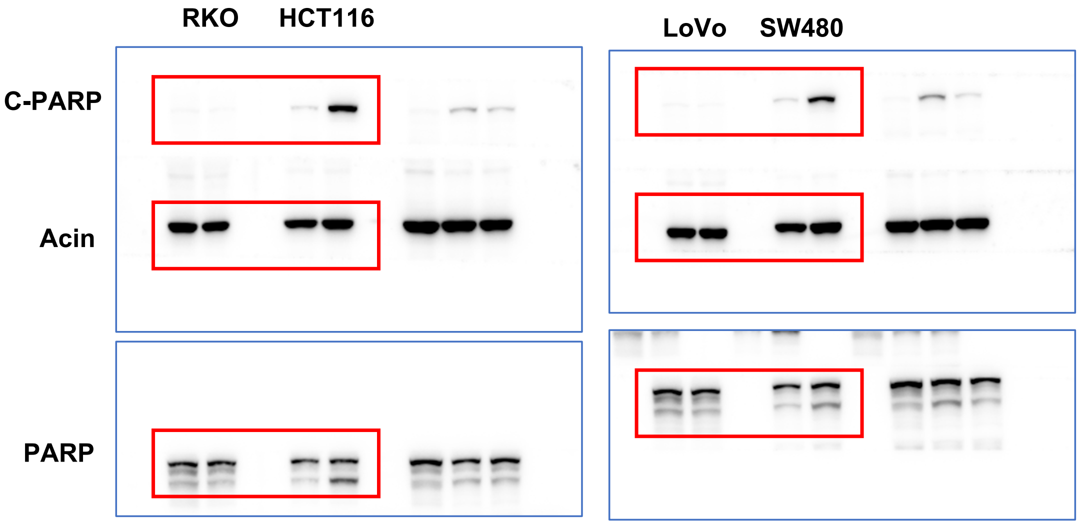

Figure 2F

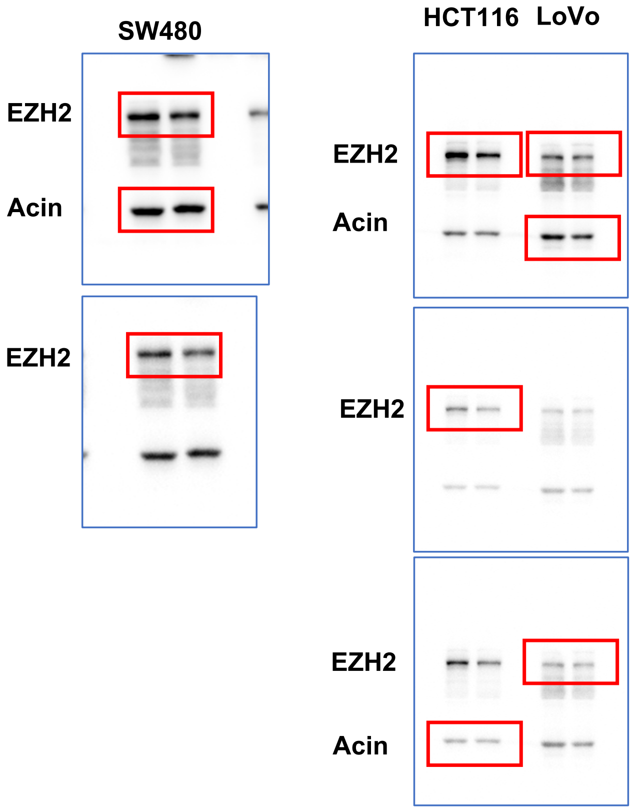

Figure 2C

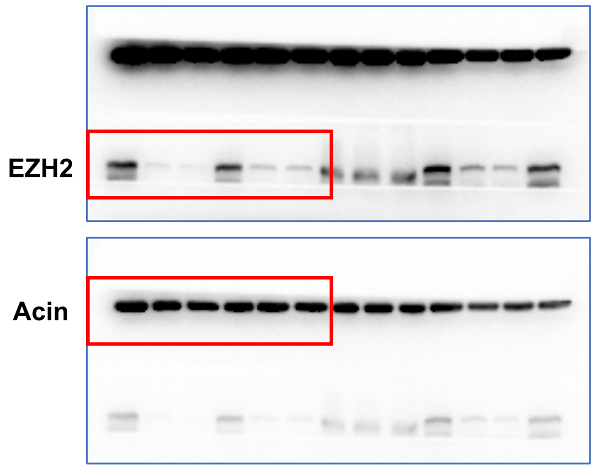

Figure 2D

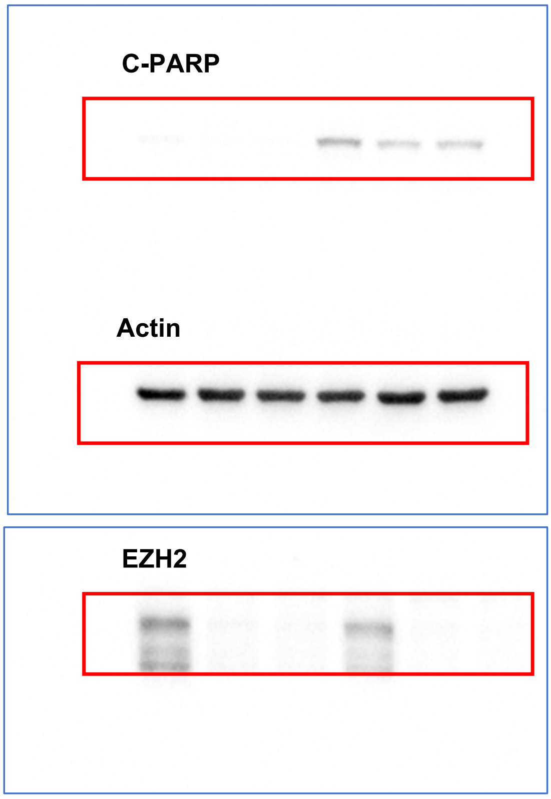

Figure 2E

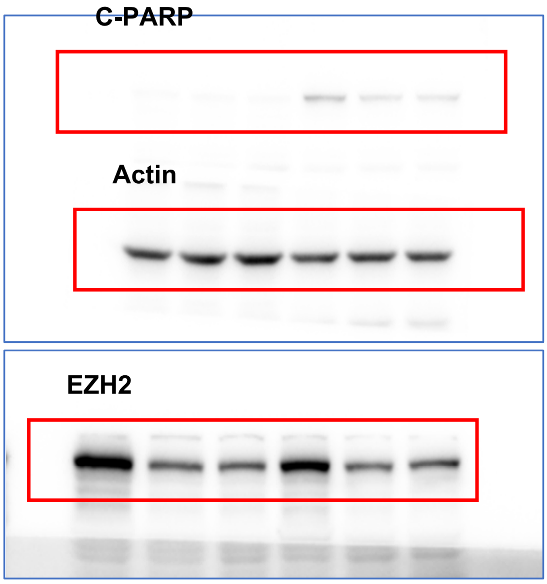

Figure 2F

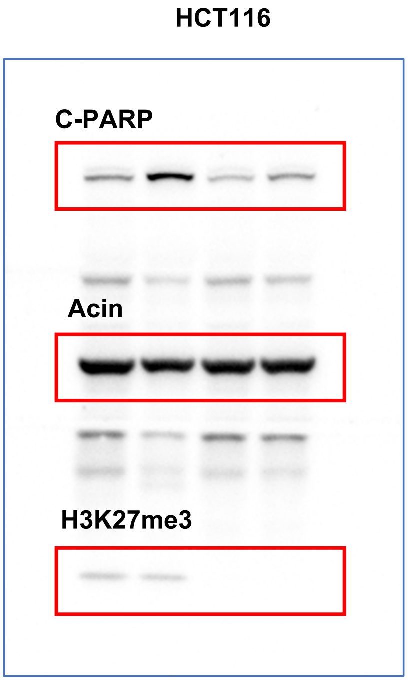

Figure 2G

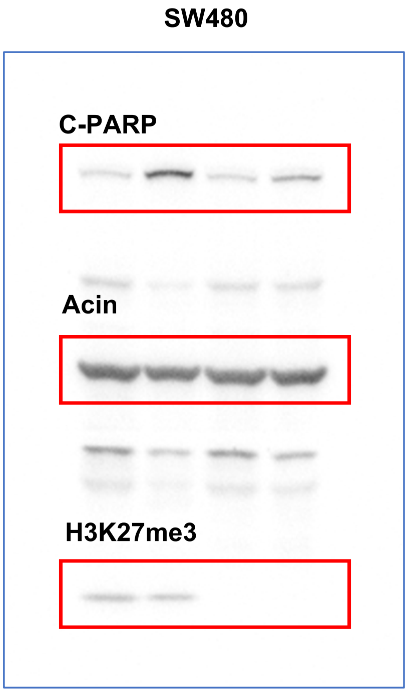

Figure 2H

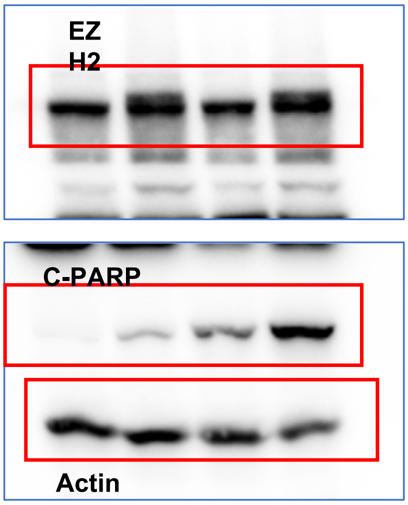

Figure 3D

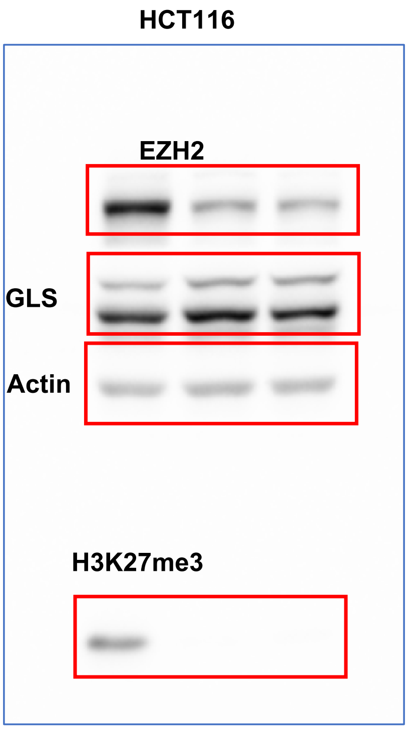

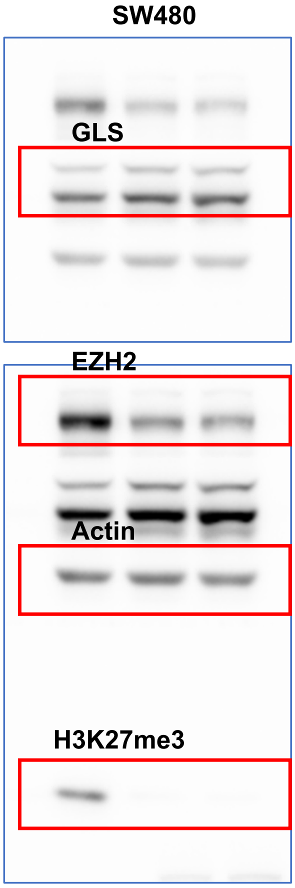

Figure 3E

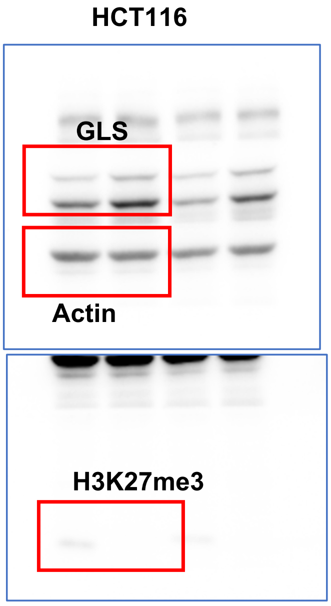

Figure 3E

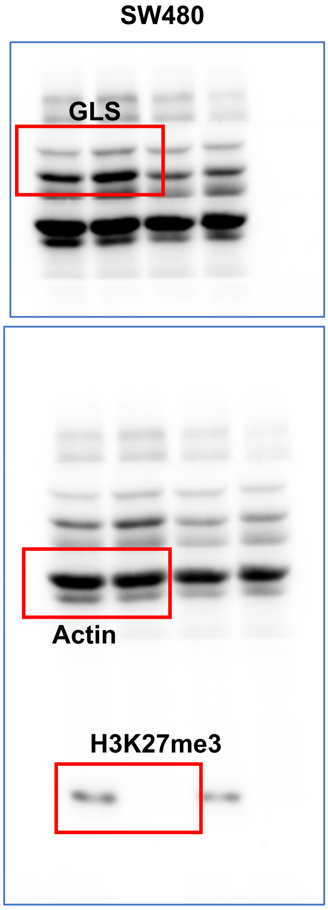

Figure 3F

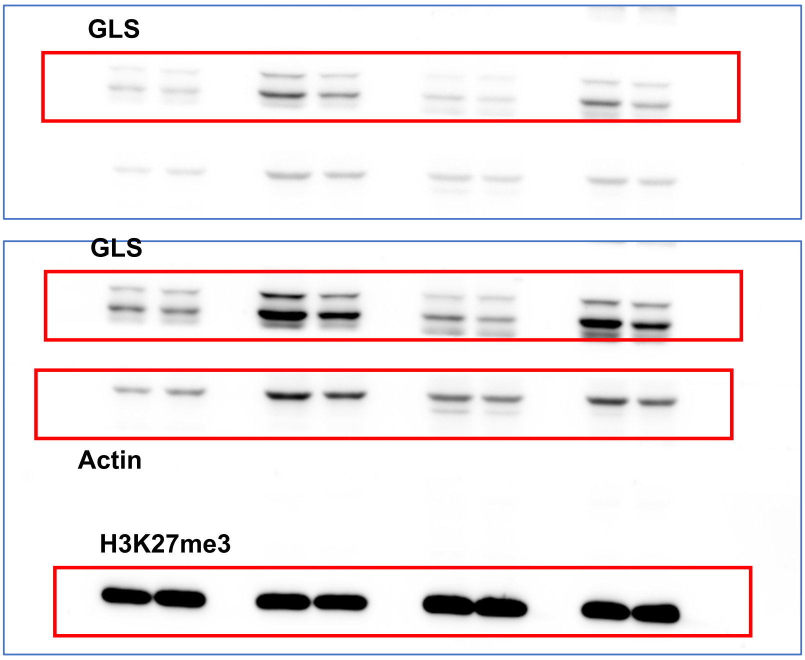

Figure 3H

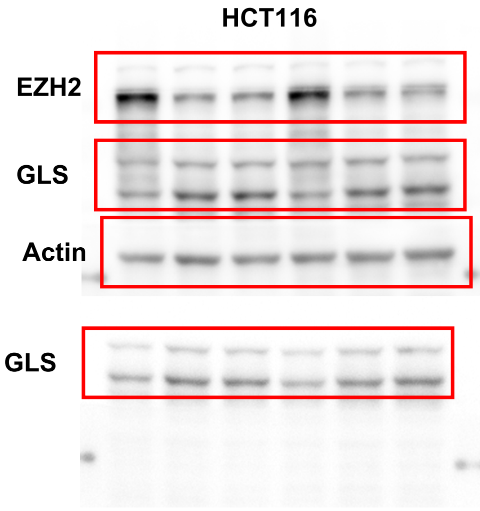

Figure 3I

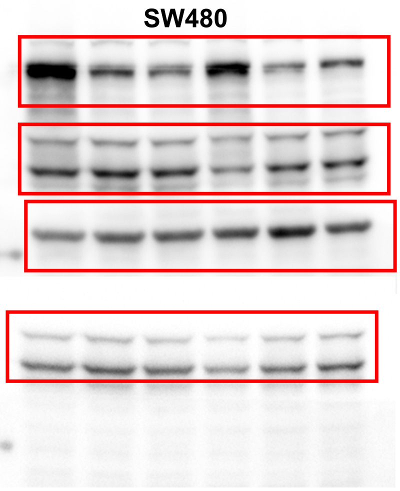

Figure 3J

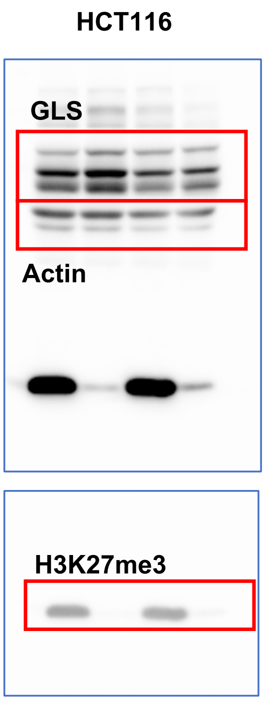

Figure 3K

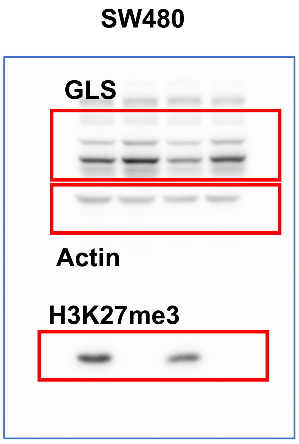

Figure 4I

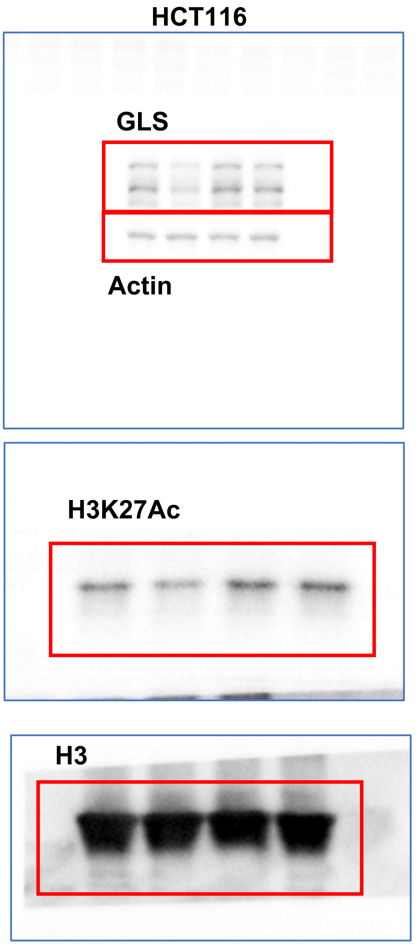

Figure 6A

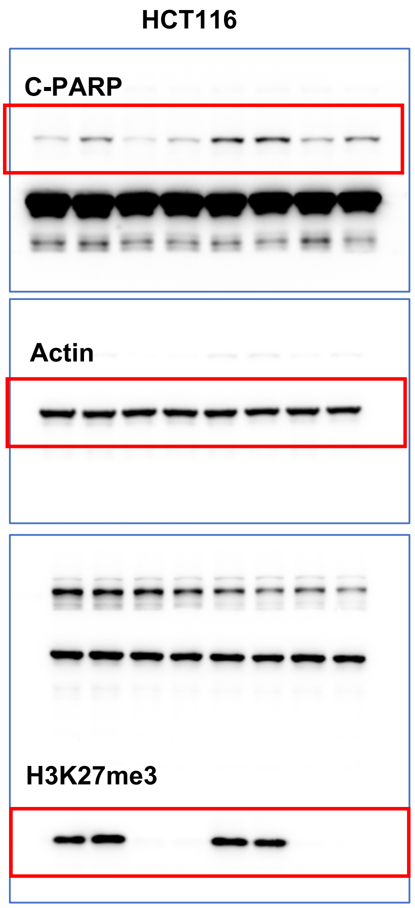

Figure 6C

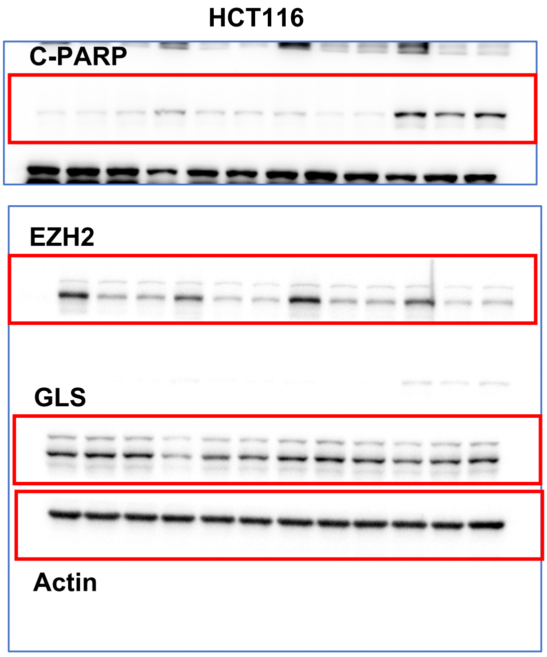

Figure 6B

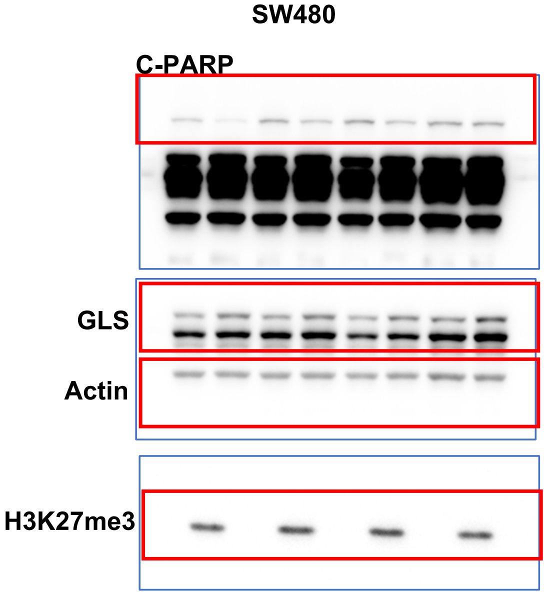

Figure 6D

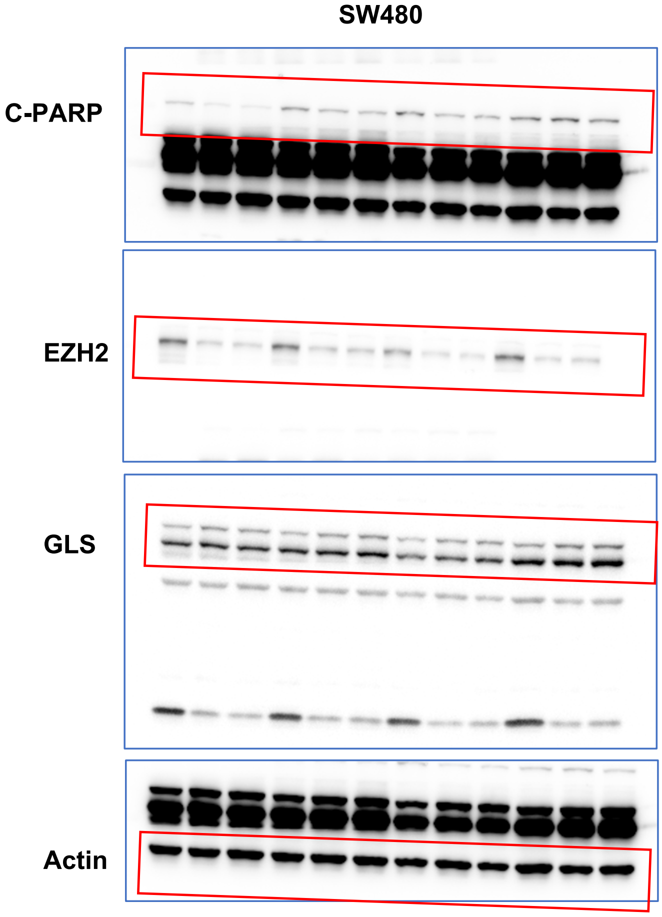

Figure 7A

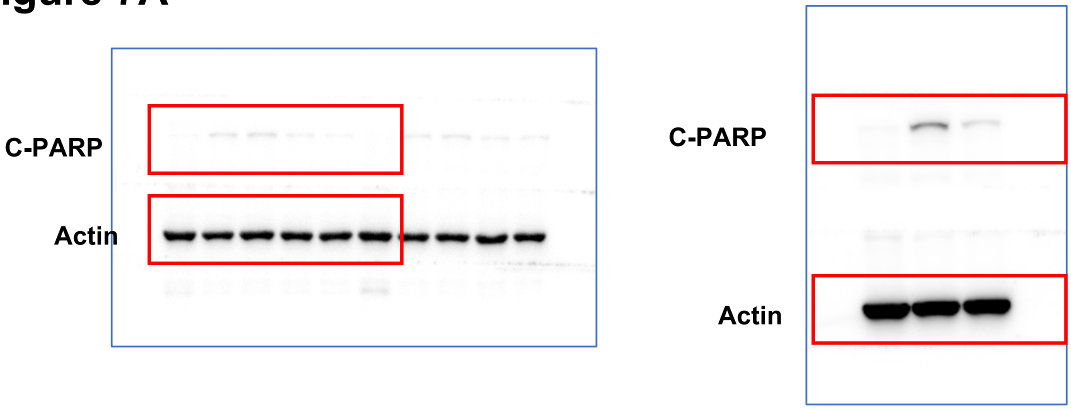

Supplymentary Figure 2B

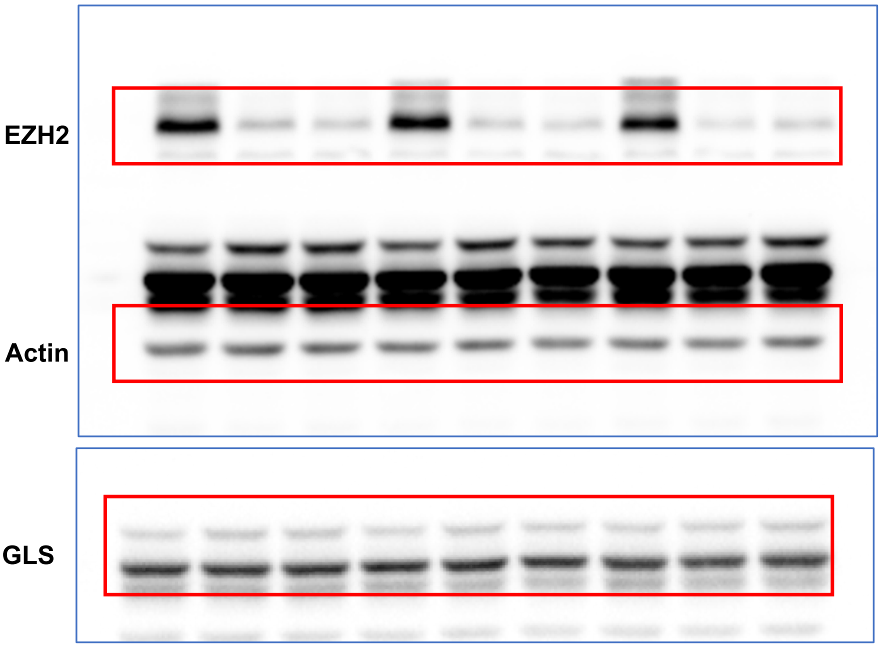

Supplymentary Figure 5B

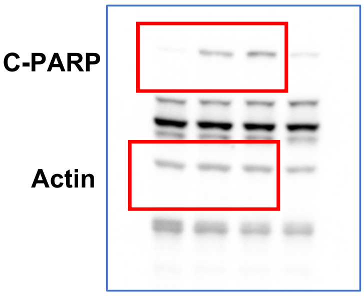

Supplymentary Figure 5C

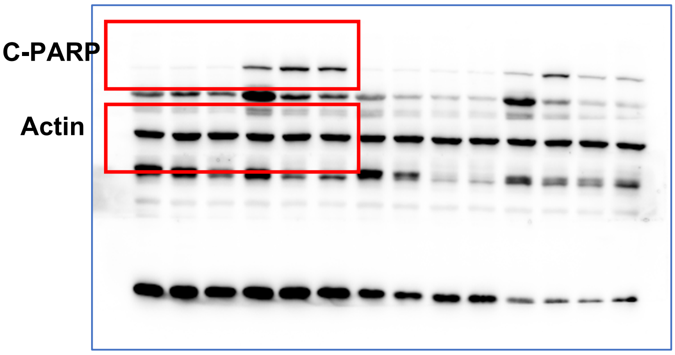

Supplymentary Figure 5D

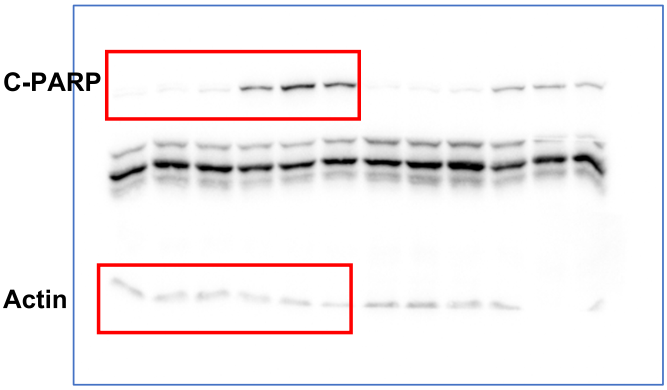

Supplement: Supplementary file 1 — Supplementary [file 41419_2021_4212_MOESM1_ESM.pdf]
